# What is the impact of nature on human health? A scoping review of the literature

**DOI:** 10.7189/jogh.12.04099

**Published:** 2022-12-16

**Authors:** Rachel M Nejade, Daniel Grace, Leigh R Bowman

**Affiliations:** 1 Department of Infectious Disease Epidemiology, Imperial College London, London, UK; 2 Abertawe Bro Morgannwg University Health Board, NHS Wales, Swansea, UK

## Abstract

**Background:**

The burden of non-communicable diseases (including poor mental health) is increasing, and some practitioners are turning to nature to provide the solution. Nature-based interventions (NBIs) could offer cost-effective solutions by reconnecting individuals with nature, but the success of these interventions depends partially on the way in which people engage with blue and green spaces.

**Methods:**

We conducted a scoping review in accordance with the Preferred Reporting Items for Systematic Reviews and Meta-Analyses extension for Scoping Reviews (PRISMA-ScR) and Cochrane guidelines to establish the evidence base for treating poor mental and physical health with NBIs. We searched five databases and the grey literature. Exposure was the active engagement with natural environments. The primary outcome was mental health and the secondary outcome was physical health defined using established metrics. All data were extracted to a charting table and reported as a narrative synthesis.

**Results:**

952 studies were identified, of which 39 met the inclusion criteria. 92% demonstrated consistent improvements across any health outcome where individuals engaged with natural outdoor environments. Mental health outcomes improved across 98% of studies while physical and cognitive health outcomes showed improvement across 83% and 75% of studies respectively. Additionally, we identified 153 factors affecting engagement with nature, 78% of which facilitated engagement compared with 22% that reduced engagement. Aspects such as the sense of wilderness, accessibility, opportunities for physical activity and the absence of noise/ air pollution all increased engagement.

**Conclusions:**

Further research (accompanied by a global improvement in study design) is needed to establish the magnitude and relative effect of nature-based interventions, and to quantify the compounding effect of factors that improve engagement with green and blue spaces. Nevertheless, this review has documented the increasing body of evidence in support of NBIs as effective tools to improve mental, physical, and cognitive health outcomes, and highlighted key factors that improve engagement with the natural world.

**Registration:**

Open Science Framework: https://doi.org/10.17605/OSF.IO/8J5Q3.

It is estimated that 10% of the global population lives with a diagnosed mental health disorder, leading to negative health and economic impacts for both individuals and the broader society [1]. Of those affected, 10%-20% are children, half of whom are already suffering from a mental disorder by the age of 14 [2,3]. Neuropsychiatric and developmental disorders such as attention deficit hyperactivity disorder (ADHD) and autism spectrum disorders (ASDs) are particularly common [4], while depression and anxiety are more prevalent among adults [1]. As individuals age into retirement, the risk of mental health illnesses increases, partly due to social exclusion, loneliness, changes to physical health, and the passing of friends and relatives [5]. If population estimates are correct, the global fraction of those aged >60 years will have increased from 12% to 22% by 2050 [5]. In the absence of effective interventions, the global burden of poor mental health will continue to climb.

In financial terms, the combined direct and indirect cost of mental health disorders across the UK in 2013 was estimated at between £70-100 billion annually [6]. Within the European Union (EU), these costs were estimated to be around €798 billion each year [7]. Worldwide, governments and international agencies such as the World Health Organization (WHO) have responded to the mental health epidemic by increasing funding for mental health research and services [8,9], yet first-line treatment for conditions such as depression, ADHD, and generalised anxiety disorder (GAD) still rely heavily on medications and psychotherapeutic treatments, such as cognitive behavioural therapy (CBT) [10,11]. Although these strategies are often effective, medications come with a long list of potential side effects [12,13], not to mention financial barriers to access [14,15]; there are also often shortages of skilled mental health practitioners to match the demand for long-term individualised CBT.

In contrast to medicated interventions, there has been renewed interest in “natural” therapies, which are seen as less intrusive and more cost-effective [16]. Meditation, lifestyle changes such as increased physical exercise, community-based activities and engagement with natural environments are emerging as potential alternatives to complement or replace other forms of treatment [16-18]. Indeed, there is growing evidence suggesting that nature-based health interventions (NBIs) can improve mental and physical health outcomes while also addressing the growing demand for less intrusive and more cost-effective treatments [16,19]. However, challenges exist; NBIs must take place in natural outdoor environments (NOEs), defined as “any environment in which green vegetation or blue water resources can be found”, access to which is becoming increasingly difficult [20,21]. Indeed, many geographical, financial, and cultural barriers affect the way we interact with NOEs, and without significant changes to the way humans live, they will likely be compounded by increasing migration away from wild spaces, and further concentration of human populations within urban areas, where 68% of the world’s population is expected to reside by 2050 [22].

Through conducting a scoping review, we aimed to set a baseline for the impact of NBIs on mental and physical health outcomes and to help with understanding the factors that magnify or diminish engagement with NOEs.

## METHODS

### Aim and objectives

We aimed to collate and assess the evidence base for NBIs and to define and assess the effect of enablers on engagement with natural outdoor environments. More specifically, we intended to locate and review the evidence base for nature-based interventions for mental and physical health outcomes, identify the enablers of, and barriers to, engagement with natural outdoor environments, and understand whether these enablers and barriers impact the effectiveness of nature-based interventions on mental and physical health outcomes.

### Study design

We conducted this scoping review according to the Preferred Reporting Items for Systematic Reviews and Meta-Analyses Extension for Scoping Reviews (PRISMA-ScR) guidelines, and the Cochrane guidelines for scoping reviews [23,24]. A scoping review was considered the most appropriate method to answer the research question, due to its capacity to answer broad questions and summarise findings from a heterogeneous body of knowledge [25].

### Study protocol

The protocol for this scoping review was drafted using the Preferred Reporting Items for Systematic Reviews and Meta-analysis Extension for Protocols (PRISMA-P) and was revised by the academic team [26]. It was disseminated through MedRxiv, the preprint server for health sciences on July 4, 2020 [19].

### Search strategy

The search includes terms relating to NBIs: a) green care, b) blue care, c) mental health, d) physical health, e) environmental determinants of NOE use, and f) socio-economic determinants of NOE use. The primary outcome of interest was mental health, defined using a number of key metrics. The secondary outcome was physical health, based on a number of physiological variables [27]. Several NBI studies have used physical health measures either as the main outcome (eg, obesity) or as an objective measure to confirm mental health outcomes obtained from self-reporting (eg, the link between stress and cortisol) [28-50]. All the keywords used for the literature search can be found in Figure S1 in the **Online Supplementary Document**.

The terminology used in the literature search for green and blue care reflects the varied positions held by researchers and the lack of consensus surrounding their application.

We used the search terms to identify studies from the following five databases: PubMed, The Cochrane Library, Web of Science, Scopus, and OVID (including Embase, PsycINFO, Global Health, MEDLINE, Health Management Information Consortium (HMIC), Transport Database). All search terms were grouped using the Boolean “OR” and were then all combined using the Boolean “AND”, to produce the final number of relevant studies identified by each database. We also performed snowballing (or the search of reference lists from included articles). To limit the effect of publication bias, we searched grey literature through Google Scholar, and governmental and institutional websites (eg, Public Health England (PHE)). Mendeley and the Covidence software were used to store, organise, and manage all references. To promote transparency and ensure reproducibility, the full search strategy used for the PubMed database is available in Table S1 in the **Online Supplementary Document**.

### Study selection criteria

The study selection was done based on the pre-defined inclusion and exclusion criteria and conducted in two stages: 1) title and abstract screening, and 2) full-text screening. If a dispute occurred on the inclusion of a study, a decision was made on the inclusion/exclusion when a consensus was achieved. We backtracked existing reviews so that any study included in both the existing review and our study was excluded from our analysis. Duplicates were removed from the search before the article screening.

As this is an emerging field, we kept the inclusion criteria for this scoping review intentionally broad. We included human studies and peer-reviewed articles on green spaces and blue spaces, with physical or mental health outcomes. Any study design was accepted. NOE exposure was based on participants’ presence in nature, whether that was confirmed through participants’ observation, interviews in nature, or through an intervention using activities in NOEs. We included any review including at least one study for which NOE exposure was confirmed by these means.

**Figure 1:**
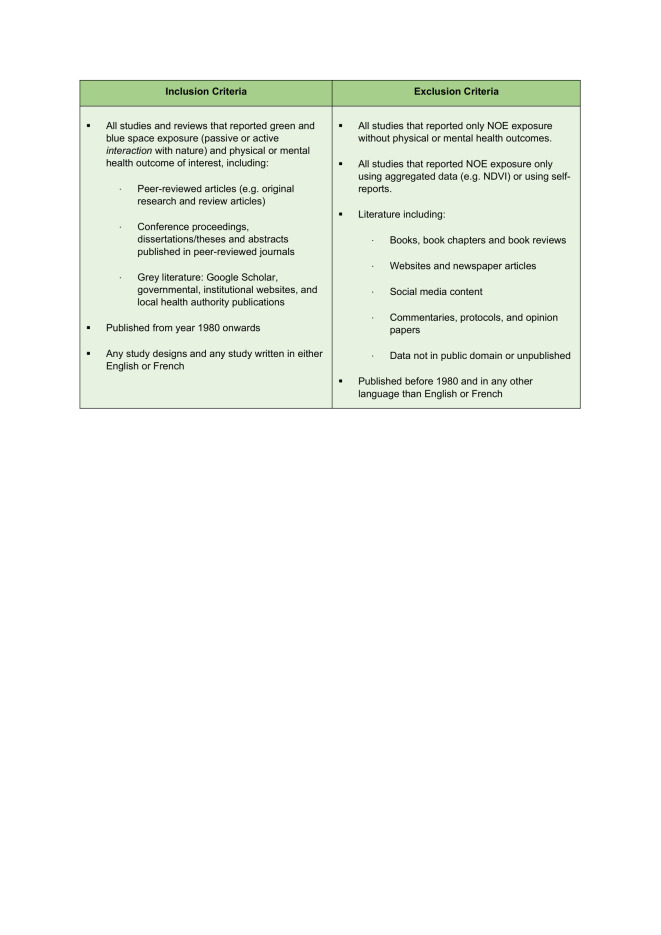
Inclusion and exclusion criteria.

Considering the contemporary topic of this scoping review, the search included all results from 1980 onwards. Studies written in both English and French were included. We excluded any studies or reviews not pertaining to health, green spaces, and blue spaces, or that were solely descriptive in nature (eg, commentaries) and studies that only defined NOE exposure based on geospatial indicators (eg, normalised difference vegetation index (NDVI)). To avoid complexities associated with recall bias, we excluded any study that used self-reported measures of engagement with nature (eg, “number of visits to parks in the last week”) [51,52]. However, this restriction was not applied to our main outcomes when these were found in studies using self-reporting scores such as GAD-7 and General Health Questionnaire (GHQ), as the validity of these measures to assess mental or physical health outcomes has been widely accepted within the scientific community. Additionally, this exclusion criterion would also have greatly reduced the number of available studies [28-50]. The full inclusion and exclusion criteria can be found in **Figure 1**.

### Data extraction and analysis

We performed data extraction (or charting) using a standardised data extraction form, adapted for this scoping review to address the research questions and objectives (Table S2 in the **Online Supplementary Document**). Content analysis was used to group findings in categories based on similarities to create a narrative synthesis of the existing evidence informed by the data charting process.

## RESULTS

### Study Selection

**Figure 2:**
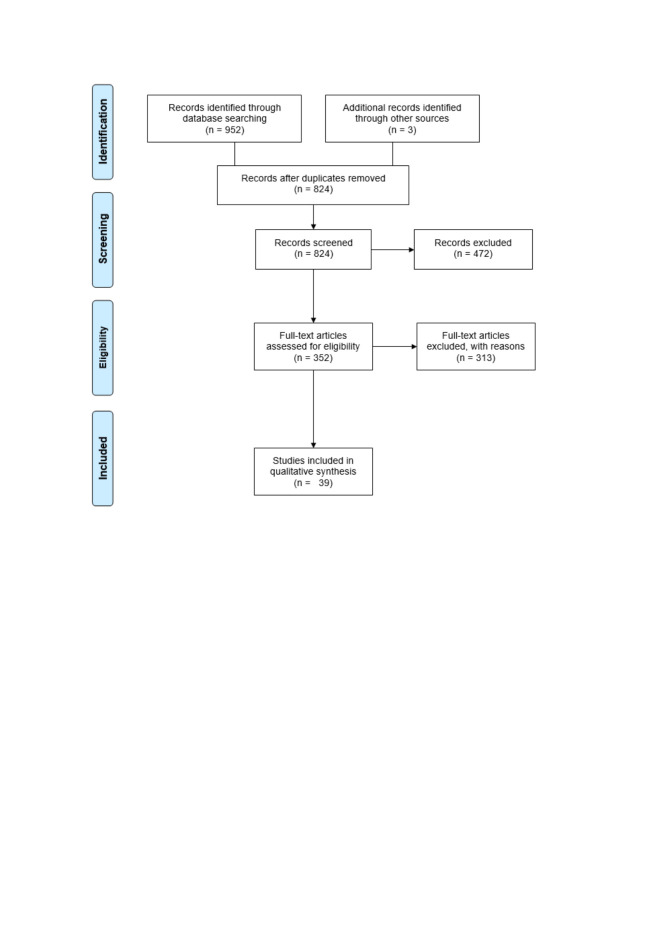
PRISMA flow diagram.

The results of the literature search across the five databases and the grey literature were reported using a PRISMA flow diagram (**Figure 2**). From the original 952 articles, 824 unique studies were identified for title and abstract screening, after the removal of 128 duplicates. Through title and abstract screening, 352 full-text articles were selected and downloaded for a full-text review (ie, eliminating 472 studies). 313 studies failed to meet the inclusion criteria at full-text screening (reasons detailed in Figure S2 in the **Online Supplementary Document**). A total of 39 articles were selected for the final analysis [53-91].

### Descriptive characteristics

A summary of each charted study can be found in Table S3 in the **Online Supplementary Document**. A total of 39 studies were included in the final analysis, 11 of which were observational, seven used qualitative methods [55-57,60,74,83,84], three used quantitative methods [63,85,88], and only one used mixed methods [75]. Among the 14 interventional studies, only one used qualitative methods [66], nine used quantitative methods [58,59,61,62,64,65,67,81,91], and four used mixed methods [53,54,87,89]. Finally, among the remaining 14 reviews, ten included systematic reviews [70-72,76-79,82,86,90], one was a scoping review [80] and three were literature reviews [68,69,73]. All studies were written in English, except for one that was written in French [82]. Additionally, all studies were carried out in the past five years, with the oldest study dating back to 2015 [57].

**Figure 3:**
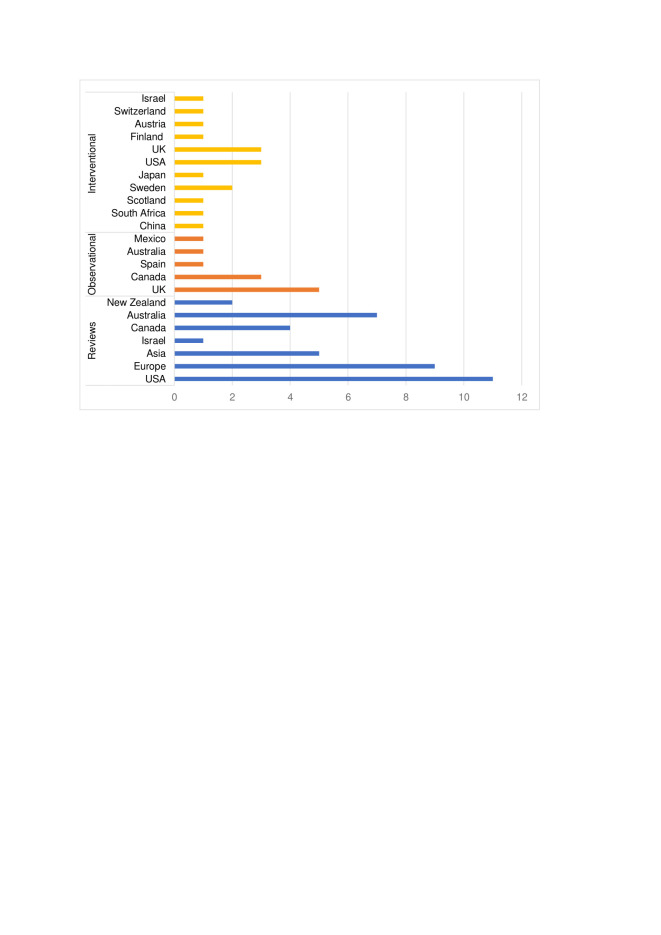
Bar chart depicting the countries included in reviews, interventional and observational studies, grouped by study design.

Most studies (85%) were conducted in higher-income countries (defined using the World Bank classification based on countries’ gross national income (GNI) per capita) [92]. Few studies were conducted in upper-middle-income countries: one observational study in Mexico [88], two interventional studies from China [67] and South Africa [61], and three reviews including Chinese [76,82] and Bulgarian studies [76,80] (**Figure 3**).

Twenty out of the 39 studies (51%) assessed the effects of engagement with NOEs on mental and physical health across all age groups, with only ten studies (26%) focusing specifically on adults (18-60 years) [54-56,58,59,62,63,67,81,91], four (10%) on the elderly (age 60+) [57,73,74,83], as well as four (10%) on children [53,66,72,88] and one (3%) on adolescents (11 to 18 years) [61].

Overall, eight studies (20%) selected participants based on age group [53,57,61,72,74,76,86,88], two (5%) based on sex (in favour of women) [62,91], and six (15%) from volunteering [54,59,63,67,81,84]. Four other studies (10%) recruited local residents [58,60,65,75]. Moreover, eight studies (20%) included patient populations with pre-existing conditions [90]. These looked at people with autism [66], neurological disabilities [73,78], mental disorders [75,84,87], or those undergoing stroke rehabilitation [64]. Notably, some studies selected participants based on their existing use of natural environments, such as regular swimmers or members of outdoor associations in blue spaces [55,56,83], or through involvement in the conservation of green spaces [89]. Finally, eight reviews (20%) did not specify any sample populations [68-71,77,79,80,82].

### Taxonomy for natural outdoor environments

Overall, three types of NOEs were identified across all studies: green spaces (51% (n = 20)), blue spaces (13% (n = 5)), and a mix of both (36% (n = 14)).

Green spaces encompassed both urban and rural environments, and most studies described green spaces as urban parks [57,62,65,69,74,82,85,88,91], natural environments [63,68,70,72,86], urban forests [53,62,78,81], or as gardens [64,73,74,78]. Other areas or features of green spaces were used less often, such as farms [53,66,78], micro-features [57,74], national parks or reserves [60,89], a game reserve [61], urban stream corridors [55], playgrounds [72], meadows [54], bogs [89], or neighbourhood greenness [77]. Similarly, blue spaces also covered urban and rural environments and were characterised by the terms: sea [56,90], blue environments [70,86], river [53], fountain/ seawall [74], coastal area [59], loch [61], wetlands [87], wilderness [90], ocean and beaches [83]. Finally, grey areas were typically considered as urban environments: urban city [54,62,65,91], built environment [58,79], urban sidewalk [59], shopping mall [62], hospital [64], urban landscape [72], roadside [81], home [91], swimming pools [83], and a field near a housing development [89].

### Nature-based health interventions

**Figure 4:**
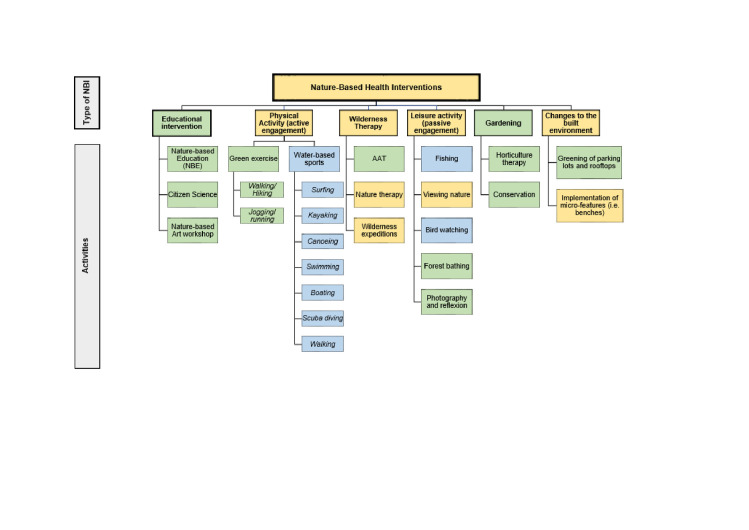
All types of nature-based health interventions found in the selected studies. Green – green spaces, blue – blue spaces, and yellow – both green and blues spaces.

All NBIs and their related activities reported across the selected studies were categorised (**Figure 4**). Six types of NBI were identified: educational intervention, physical activity, wilderness therapy, leisure activity, gardening, and changes to the built environment.

### Health outcomes and nature-based interventions

All reported outcomes and their associated enablers are listed in Table S4 in the **Online Supplementary Document**. Almost all of the studies included at least one mental health outcome [53,54,56-66,68-87,89-91], except for three that focused solely on physical activity [55,88] and cardiovascular outcomes [67]. Many studies used multiple outcomes, and each of these is reviewed and discussed in the following order: mental health outcomes, physical health/physiological outcomes, and cognitive health outcomes.

**Figure 5:**
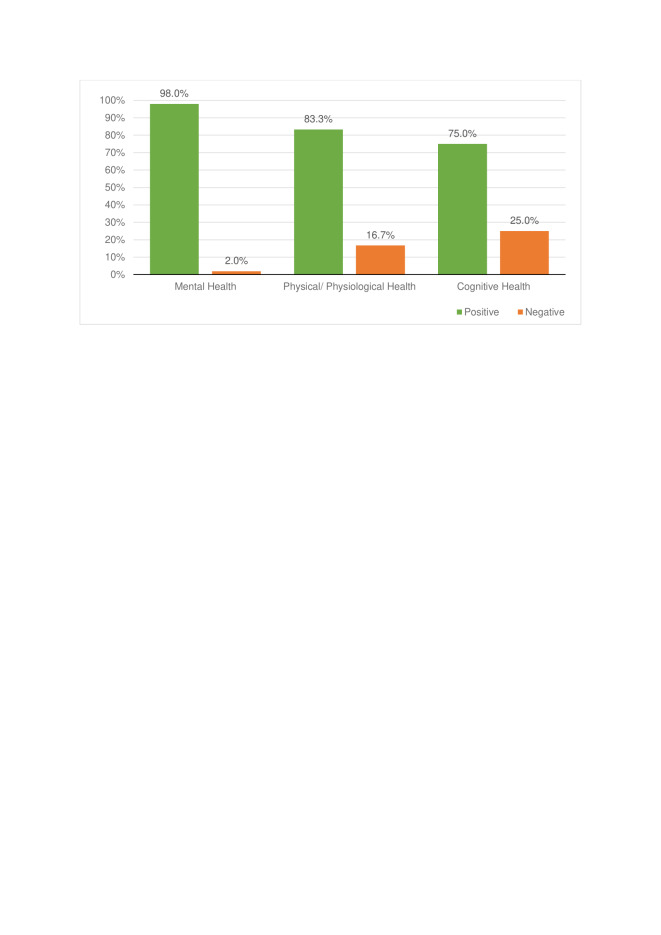
Percentage of positive and negative findings stratified by health outcomes.

Overall, there are clear positive trends between NOE engagement (through voluntary participation or primary care intervention) and psychological, physical, and cognitive health outcomes (described in **Figure 5** by the bars labelled “positive findings”). In applicable studies [56,73-75,83,87], a decrease in the measurable outcome was considered a “positive finding” where this resulted in a gain for the individual eg, a reduction in social isolation. The studies displayed as “negative findings” refers to studies where health outcomes led to mixed or no positive effects [59,70,71,76,81,82,87,91].

### Mental health

Mental health was the most commonly studied outcome (62%). There were improvements across all mental health outcomes when engaging with nature (98%), with only one study reporting no effect (2%) [71]. No negative effects were found.

Engagement with NOEs led to an improved quality of life in 4% of all studies looking at mental health outcomes, as assessed by measures of Health-Related Quality of Life (HRQoL) [53,64] or Quality of Life (QoL) surveys [69,86]. Only one study reported improved “perceived mental health” (ie, restoration) of stream-corridor users, assessed using qualitative interviews [57]. Outcomes related to measures of well-being were the most studied ones and were usually positively associated with NOE engagement. It was measured differently across studies and relied on measures of hedonic and eudaimonic well-being [71], perceived well-being [56,73-75,83,90], and general well-being [54,58,63,68,71,76,86,87]. Only one systematic review reported mixed effects, which the authors attributed to poor study design and quality [71]. Finally, measures of depression [63,65,78,80] and anxiety [64,65,78,81,87] decreased when engaging with NOEs.

There was also a positive effect of NOE engagement on measures of emotional health outcomes across all studies. Most reported improved affect [58,64,70,73,81-83,86,87], mood [62,65,79,80,89,91], self-esteem [61,73,80,84,90], self-confidence [75], and vitality [62,66]. Others reported decreases in negative affect [63,81,83,86], mood disturbances [65], agitation [73,78], and behavioural problems (eg, hyperactivity or violence) [72,73,80,82].

Overall, engagement with NOEs led to improved social health across 100% of the fourteen studies that assessed their effects. Six studies reported reduced social isolation [56,73-75,83,87] and one found reduced social discomfort [91] following engagement with NOEs; seven noted increased social connectedness between individuals [66,68,78,82-84,90].

Finally, several studies assessed the effects of engagement with NOEs on stress. All studies reported positive associations with psychological resistance [54,56,90], perceived restoration [59,60,62,65,82,91], and stress reduction [54,63,66,73,81-84,89]. Only one study found a decrease in psychological distress [80], and three found decreases in perceived stress [63,86,87], which all translated into health benefits.

### Physical/physiological health

83.3% of the studies considering physical and physiological health outcomes found benefits across a range of outcomes; 16.7% yielded no or negative effects for measures of obesity [70,76,82,87], heart rate [65], systolic and diastolic blood pressure (BP) [67], and heart rate variability (HRV) (2%) [79].

All measures of physical activity in natural environments demonstrated that engaging in NOEs led to increased physical activity. This was measured in several ways. Some studies used measurements of leisure-time physical activity [55] or reported use after urban green spaces interventions [69,74,82]. Others focused on increased exertion post-engagement with NOEs, using measures of moderate to vigorous physical activity (MVPA) [79,88]. Similar methodologies used measures of perceived physical activity [56,57,68] and physical fitness [90], or more broadly an increase in the use of NOEs for various activities like swimming [56] or walking in nature [60,76]. Finally, decreasing sedentary time was used as a measure in children populations [88].

One systematic review assessed the effect of engagement with green spaces on sleep during a walking intervention and found that engagement led to improvements in sleep quality and quantity [77]. Similarly, one study reported improved recovery from mental disorders after engaging in therapeutic horticulture as part of a recovery program [75].

Motor functioning was assessed differently by two studies [64,74]. Ottoni et al. [74] reported improved mobility after walking interventions in green spaces, while Pálsdóttir et al. [64] reported reductions in disability after engaging in horticulture therapy for post-stroke patients. Overall, improvements in disability were reported in both intervention and control groups, suggesting that the therapy itself may facilitate recovery more than the type of environment [64].

All studies measuring physical health outcomes found a positive association between physical health and NOE engagement when measured by GHQ [72,80,87]. Pálsdóttir et al. [64] used post-stroke fatigue (PSF) as their main outcome, which decreased following horticulture therapy. Importantly, both the intervention and control groups experienced decreases in PSF, thereby reducing the importance of the intervention in this context over other mainstream standards of care.

Four studies reported little to no effects on obesity (measured using body mass index) after engagement with NOEs [70,76,82,88]. Regarding mortality, only two studies investigated how NOE engagement affected all-cause mortality [70,79]. Both studies found a decrease in mortality following changes to the built environment [70] and after engaging in physical activity in nature [79].

Cardiovascular health was measured using diastolic and systolic BP [62,65,67], baseline resting heart rate [54,65,67,69], and HRV [62,79,91]. Heart rate was found to decrease in 80% of studies looking at this measure, except for one [65]. Similarly, BP was found to decrease in three studies, except for one by Ana et al. [67], which found no changes in BP after forest bathing. Results were also inconclusive for HRV, which tended to increase after NOEs exposure in two studies [62,91], but had no effects in another [79].

Physiological measures of stress were determined using cortisol samples; in two studies, there was a decrease in cortisol levels after engaging in NOEs [62,82].

### Cognitive health

Although not initially included, cognitive health outcomes were identified on several occasions (8%) during the analytical process and were considered important for this review. Overall, NOE engagement had positive effects on cognitive health (58%), by reducing ADHD symptoms (8%) [72], and by improving cognitive functioning (50%) [53,54,66,72,79], except in one study (8%) [59]. Findings on memory were inconclusive (32%) [72,78,81,91].

Cognitive functioning was reported using measures of science, technology, engineering, and math (STEM)-capacity [53], attention restoration [54,72], and attention retention [59,66,79,82]. In 86% of these studies, cognitive functioning was positively associated with NOE engagement, except for one study which reported no change in attention retention [59]. However, attention retention was improved after exposure to natural environments in three studies [66,79,82], along with attention restoration [54,72]. One study also showed an improvement in children’s STEM-capacity following a nature-based education (NBE) intervention [53].

Memory was only assessed in four studies and yielded mixed findings [72,78,81,91]. While one found a positive association between spatial working memory and engaging in NOEs [72], the other found no effects [91]. Similarly, for executive functioning, one study found no effects [81], while the other saw improvements in executive memory [78].

During a wilderness expedition, trained therapists noticed a decrease in ADHD symptoms for children living with autism after exposure to animals and the natural environment [66] – which was supported by McCormick [72] in her systematic review.

### Engagement with natural outdoor environments

**Figure 6:**
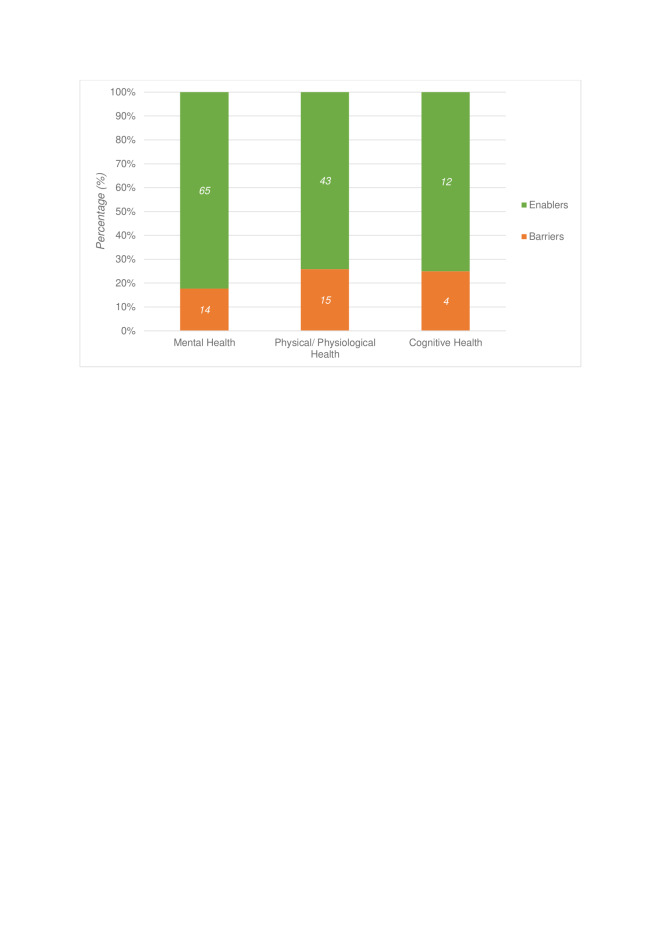
Number and percentage of enablers and barriers for each health outcome. Enablers in green; barriers in orange.

Several factors influencing engagement were identified throughout the selected studies. These factors were divided into those that facilitated engagement (enablers ( ~ 78%)), vs those that hindered engagement (barriers: (22%)) (**Figure 6**). These included environmental, social, individual, and structural processes, along with opportunities for physical activity and stress reduction. Poor study design and quality were considered barriers across all studies. A description of each enablers’ category can be found in Figure S3 in the **Online Supplementary Document**.

### Environmental processes

Most enablers focused on environmental processes (38%), the most common being the type of environment (66%), where natural environments facilitated health benefits over built environments (ie, swimming pools, city centres, shopping malls, etc.) [53-56,58,59,62,63,65-67,70,72,73,77-84,86-91]. Although the variety of green space and blue space descriptors makes comparing studies difficult, some studies have found that urban forests were better than urban parks, as they reduced cortisol levels [62], BP [54,62,65], and heart rate [62,67,79], while increasing HRV [62,91]. Interestingly, one study even found that heart rate benefits were amplified if that forest was made of maple trees as opposed to birch or oak trees, while BP would not change if the temperature, humidity, and light spectrum (ie, green/blue light ratio) were too high [67]. Similarly, blue space users preferred wilder and more available water environments (eg, ocean) as they amplified psychological health benefits through increases in well-being and social health benefits by reducing social isolation [56,83].

Typically, biodiversity was shown to facilitate well-being [87], psychological restoration [85], social connectedness [87], positive affect [83], and overall health [87], while reducing anxiety [87] and stress [87]. Notably, biodiversity may also present a barrier if perceived as threatening or harmful (for example, due to the presence of sharks in blue spaces [83]). Other environmental processes such as good weather [83], heat reduction [70], seasons [65], perceived aesthetics [68], nature connectedness [58,71], the presence of farm animals for autistic children [66], and sensory qualities of the environment (ie, sound) [59,60] were all found to also improve mental, physical, physiological and cognitive health; however, other detrimental environmental processes such as air and noise-related pollution [62,76] negated these positive effects.

### Structural processes

Structural processes were the second most common enablers discussed in this scoping review (37%). Among them, good accessibility was most commonly reported (24%), as it facilitated improvements in perceived mental health [57], overall health [72], positive affect [70], physical activity in NOEs [55,57,74,82], and attention restoration [72], while reducing social isolation [74], motor disability [74], behavioural problems, and psychological distress [80]. Similarly, geographic proximity to NOEs was also mentioned several times (11%) as facilitating well-being [76], physical activity [60,69,76], cognitive functioning and spatial working memory [72].

The type of intervention was also reported by six studies (16%) as facilitating the health benefits gained from engaging in NOEs. Britton et al. [83] and Ottoni et al. [90] recognised that activities in blue spaces, such as surfing or swimming, contribute to rehabilitation, stress reduction, and health promotion, and complementary evidence demonstrates that therapeutic horticulture led to improvements in PSF [64] and reductions in agitation for older adults [74]. Additionally, viewing nature decreased BP [62] and improved executive memory [78]. Interestingly, the outcomes improved with increases in the length of the activity [53,61,87,88]. One study found that activities performed in the afternoon instead of the morning improved sleep quality and quantity, believed to be caused by a two-process model where sleep and waking are regulated by circadian rhythms and homeostasis [77,93]. Good group organisation, transportation, and staff attitudes and knowledge were also considered enablers of the associations between health and nature [87]. However, when NBIs have limited resources, the strength of these associations is reduced [73,90], and hence, good NBI quality and design can amplify the health benefits gained from nature.

The quality and design of NOEs were also found to amplify health benefits when engaging with nature, as the presence of micro-features of the environments (eg, benches) was found on several occasions to improve well-being and self-esteem while reducing social isolation and stress in individuals with dementia [73]. Older adults also found that benches could help decrease social isolation [74] and improve their mobility and physical activity in NOEs [74]. Other studies also found general increases in physical activity and positive affect when these features were present [55,70,80]. Overall, positive changes to the environment through the implementation of micro-features were found to facilitate engagement in NOEs.

### Individual processes

Most individual processes across the selected studies were considered barriers (74%) as opposed to enablers (26%).

Safety concerns were the most common barriers to engaging in NOEs (24%), as they worsened perceived mental health [57], positive affect [70,73], perceived restoration [60], physical activity [55,57], well-being and self-esteem [73] while increasing social isolation and stress [73]. Stigma was another recurrent barrier found across studies (12%) that diminished perceived well-being [73,90], physical activity [56], physical fitness, social connectedness and psychological resistance [90], as well as positive affect and self-esteem [73], while increasing social isolation [73] and stress [73].

Other barriers such as social prejudice [73], fear [56,90], negative self-perceptions [57,73], poor self-confidence [73], individual factors (eg, time pressure, changing identities) [74,77], and deprivation [80,84] were also detected. Conversely, some individual processes were found to facilitate the relationship between nature and health. These included cognitive functioning [72], some intrapersonal processes (ie, individual preferences) [68], gender (whereby women tended to benefit more than men) [61,74], and age (since younger adults and children had increased health benefits from engaging in NOEs due higher engagement in physical activity than older adults) [82].

Lower socio-economic status (SES) and ethnicity were identified as both enablers and barriers. While one study found that being South Asian and living in the UK led to worse health outcomes than being British white [80], another found that Arab women benefited more than Jewish women when engaging in NOEs [91]. The latter was thought to be influenced by levels of comfort at home, where Jewish women reported feeling more comfortable in their home than Arab women did and therefore gained fewer marginal improvements than Arab women when engaging in NOEs [91]. Similarly, lower SES was found to increase health gains through NOE engagement [82], whereas another found it led to worse health outcomes [76].

### Opportunities for physical activity

Opportunities for physical activity were the third most frequent enabler found across studies (11%). They included physical activity (72%) and active engagement in NOEs (18%), as both were found to magnify the benefits for mental health [56,58,63,71-73,75,79-82,89], physical health [56,72,75,80], physiological health [79], and even cognitive health [72,78,79]. However, these benefits would be reduced if participants were injured or had mobility difficulties [74,78]. Physical activity could therefore be another mechanism by which nature positively influences health.

### Social processes

Social processes were not as common as other enablers (7%), but were found to influence the nature’s impact on health. The presence of other people was the most common enabler (29%) and barrier (29%) across studies considering social processes. Indeed, two studies reported that sharing the experience of engaging in NOEs with others could facilitate gains in physical activity [55], recovery from mental disorders [75], social connectedness, self-esteem, and self-confidence [84] while reducing social isolation [75]. However, if other individuals were perceived as safety risks, well-being and physical activity would decrease, while stress would increase [63].

Additionally, social interactions, interpersonal processes, group membership, and the presence of caregivers also facilitated positive gains in psychological [68,78,89], social [68,83] and physical health [68,89]. Therefore, social processes are other mechanisms through which health benefits can be gained from nature.

### Opportunities for stress reduction

Despite abundant evidence from the literature review, only 1% of all enablers focused on opportunities for stress reduction. Stressful life events were perceived as barriers, as they decreased the quality of life, well-being, positive affect, psychological resistance, and STEM capacity for children [52,63], while worsening depression in adults [63]. However, engaging in NOEs was shown to reduce stress in all studies looking at stress-related outcomes, considered measures of psychological health in this review [53,63,66,73,81-84,89]. Therefore, evidence for stress reduction as a mechanism in the relationship between health and nature is moderate, but not as conclusive as other enablers.

### Study quality and design

Methodological choices when conducting studies (9%), such as the study design (44%), study quality (44%) or the choice of measurements (12%) were all found to negate the relationship between health and nature across selected studies [59,70,71,76,81,82]. They were responsible for the lack of evidence between NOE engagement and obesity [70,76,82], well-being [71], HRV [79], and on measures of memory [81] and cognitive functioning [59]. Therefore, the methods used within studies also act as potential mechanisms on nature and health.

## DISCUSSION

**Figure 7:**
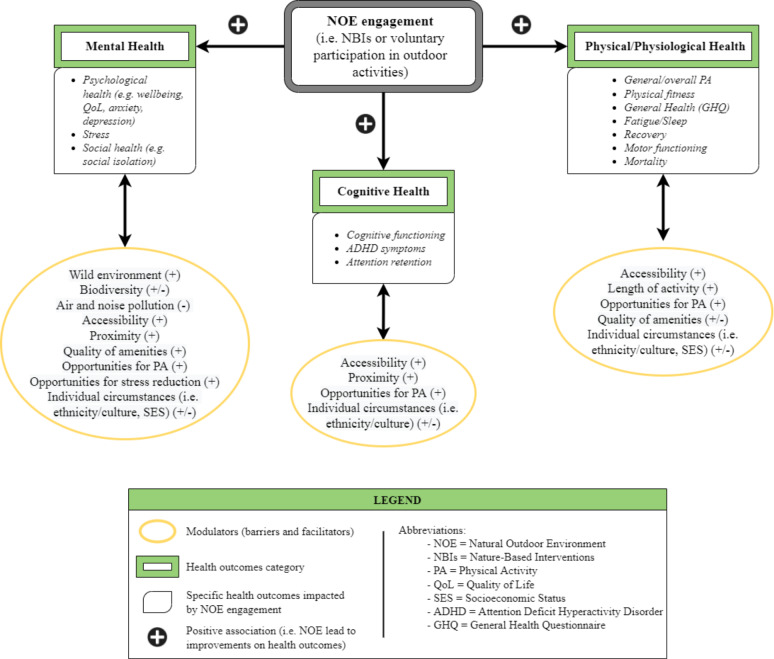
Impact of natural outdoor environment (NOE) engagement and enablers on health.

This scoping review synthesised heterogeneous research documenting the impact of nature on health. Of the 39 included studies, nature-based interventions were found to have improved mental, physical/ physiological and cognitive health outcomes across 98%, 83%, and 75% of articles, respectively (**Figure 5**). Furthermore, this study identified a breadth of factors that affect the level of engagement with NOEs, and by extension the likely success of nature-based interventions (**Figure 7**).

### Nature-based interventions and health

As a species, humans have become increasingly sedentary. Offices, schools, homes, and public spaces have been designed to optimise and prioritise efficiency. At least in part, this relatively new lifestyle (by historical standards) is driving an increase in non-communicable diseases, including poor mental health [94]. As individuals continue to seek work in urban areas, the opportunity to interact with green and blue spaces diminishes. Current estimates indicate that over 50% of people worldwide live in urban areas projected to increase to >68% by 2050 [22,95].

Considering this, it is not surprising that the reintroduction of nature into a person’s life, irrespective of baseline physical and mental health characteristics, can have a positive influence [96]. Research shows that individuals living in urban areas with more green space have both lower mental distress and higher well-being scores [97]. “Forest-bathing” (“shinrin-yoku” in Japanese) in Japan has been shown to significantly lower salivary and serum cortisol levels when compared to control groups [98], while Niedermeier et al. [99]f ound that hiking resulted in a statistically significant increase in “affective valence” (ie, pleasure) when compared to a sedentary control group and an indoor exercise group.

One theory that might begin to explain these mechanisms is that, when in natural outdoor environments, individuals experience a reduction in “rumination” – a maladaptive pattern of self-referential thought that is associated with heightened risk for depression and other mental illnesses [95]. Indeed, data suggest this might be plausible: functional magnetic resonance imaging (MRI) scanning performed on individuals who had spent 90 minutes on a nature walk showed reduced neural activity in the subgenual prefrontal cortex (sgPFC) – an area of the brain that displays increased activity during sadness and rumination. However, participants who went on an urban walk did not show these effects [95].

Such data makes clear the physiological responses that NBIs elicit in humans, and while further granular data are required, the mounting body of evidence generally supports nature-based interventions for the prevention and treatment of physical/ mental health ailments. Indeed, science is beginning to inform public health policy via the introduction of “green prescriptions”, which are clinically prescribed NBIs for treating physical and mental health disorders [100,101].

The broad evidence base uncovered by this scoping review demonstrates the positive impact of NBIs on mental, physical, and cognitive health outcomes. Indeed, the findings support national policies that integrate NBIs as effective preventative and curative tools for public health [16,19,100,101].

### Factors impacting engagement with natural outdoor environments

#### Biodiversity and wilderness

Our findings on the importance of biodiversity and wilderness as drivers of impactful NOE engagement provide support for a broader interconnectedness between humans and wild spaces. This applies to all projects at any scale, from school expeditions through urban greening to broader rewilding. Enabling interaction with NOEs through accessibility (both geographic proximity and improved infrastructure) magnifies the health benefits of NOEs [55,57,60,69,70,72,74,76,80,82] and facilitates interaction between the public and natural ecological systems [102], promoting greater understanding and awareness of nature’s importance. The creation and maintenance of long-distance trails [102], increasing the sense of “wild” in urban green spaces [83,85,87], and a departure from meticulous park management [55,70,73,74,80] are examples of practices that result in increased “quality”, accessibility, and biodiversity, leading to plausible health gains through greater NOE engagement [55,70,73,74,80,83,85,87,102]. This recommendation fits within the broader International Union for Conservation of Nature (IUCN) vision for human interactions and ecosystem health to “[…] protect, sustainably manage, and restore natural or modified ecosystems, that address societal challenges effectively and adaptively, simultaneously providing human well-being and biodiversity benefits” [103].

#### Air and noise pollution

Our findings also support wider initiatives targeting reductions in air and noise pollution, as these were found to negatively impact the time that users would spend practising physical activities in NOEs [62,76]. Cleaner, greener environments would also encourage physical exercise and contribute to national and global targets to mitigate climate change [62,76,104]. Indeed nature-based initiatives, such as de-pollution and re-naturalisation of urban sites, are currently under consideration by the EU Commission as methods to achieve an increase in the number of publicly available green spaces, and reverse social inequalities [104,105].

#### Socio-economic status and stigma

Cultural and ethnic differences, as well as deprivation, were found to limit the health benefits gained from engagement with NOEs. Minority groups living in more deprived areas with poorer access to, and lower quality of, green spaces, had more behavioural difficulties than non-minority groups [80,91]. Despite mixed findings in this review [76,82], existing inequalities concerning access to urban green infrastructure remain, along with inequalities in the exposure to health hazards (eg, air and noise pollution), particularly for vulnerable groups such as children, the elderly, and individuals of lower socio-economic status [106]. These inequalities are well-documented in urban areas across many European countries and likely exist globally, highlighting the need for urban greening initiatives that work towards reducing social barriers to access, and increasing the use of green and blue environments [106,107].

#### Geographic proximity and opportunities for physical activity

The sedentary lifestyle characterising modern society has also led to a clear reduction in physical activity across age groups [102]. As regular physical activity has been shown to reduce certain health risks (such as cardiovascular diseases or symptoms of depression and anxiety), health agencies such as the WHO have urged governments to promote physical activity to their populations as a way to limit the growing burden of ill health [27,108].

The results from this review support the need for enhanced engagement in physical activity, especially when practised in green or blue environments, as these environs likely magnify the mental, physical and cognitive gains. Importantly, structural enablers such as good accessibility [55,57,74,82] and closer geographic proximity to NOEs [60,69,76] led to increased physical activity. This is important for policymakers, as it highlights the need to consider access and proximity to green and blue spaces when designing health interventions that promote physical activity.

### Limitations

Methodologically, the exclusion of studies based on self-reported measures of exposure (eg, number of visits in the last month) could have precluded the inclusion of additional relevant studies to this review. However, this was deemed necessary to limit the inherent risk of recall bias in these studies, which could have impacted the strength of the results. The absence of critical appraisal of individual sources of evidence precluded the possibility for our results to lead to statistically significant conclusions. Nevertheless, scoping reviews as per PRISMA-ScR guidelines do not necessarily require a critical appraisal of the evidence for structural integrity; as a minimum, they promote a stronger evidence base [23].

The comparison between health outcomes and types of green spaces or blue spaces was made difficult due to the variety of terms used to describe these areas. Similarly, for nature-based interventions, direct quantitative comparisons were difficult due to the absence of magnitudes, relative effects, varied heterogeneous study designs, and sample sizes.

## CONCLUSIONS

Further research is still needed to establish the magnitude and relative effect of nature-based interventions, as well as to quantify the compounding effect of factors that improve engagement with green and blue spaces. This must be accompanied by a global improvement in study design. Nevertheless, this review has documented the increasing body of heterogeneous evidence in support of NBIs as effective tools to improve mental, physical and cognitive health outcomes. Enablers that facilitate greater engagement with natural outdoor environments, such as improved biodiversity, a sense of wilderness, and accessibility, as well as opportunities for physical activity and an absence of pollution, will likely improve health outcomes and further reduce public health inequalities.

## Additional material


Online Supplementary Document
