# Supplementary material for: What is the impact of nature on human health? A scoping review of the literature
Source: J Glob Health. 2022 Dec 16;12:04099. doi: 10.7189/jogh.12.04099 (PMC9754067; doi:10.7189/jogh.12.04099)
Supplement: Online Supplementary Document [file jogh-12-04099-s001.pdf]

ONLINE SUPPLEMENTARY DOCUMENT

Title: What is the Impact of Nature on Human Health?: A Scoping Review of the Literature

Authors: Rachel M Nejade,[1] Daniel Grace,[2] Leigh R Bowman,[1*]

Affiliations
1. Department of Infectious Disease Epidemiology, Imperial College London, St. Mary's Campus, Norfolk Place, London W2 1PG
2. Abertawe Bro Morgannwg University Health Board, NHS Wales

*Corresponding author, Leigh Bowman

Date 28 Oct 2022

Green care: 'green care', 'green prescriptions', 'nature-based interventions', 'ecotherapy', 'green skills', 'green social care', 'green health', 'outdoor therapy', 'nature therapy', 'nature-based therapy', 'green therapy', 'social prescribing', 'green exercise', 'green spaces', 'green interventions'
Blue care: 'blue care', 'blue prescriptions', 'blue prescribing', 'nature-based interventions', 'blue therapy', 'blue activities', 'blue exercise', 'blue spaces', 'blue interventions', 'blue skills'
Primary health outcome (mental health): 'mental health', 'wellbeing', 'life-satisfaction', 'stress', 'anxiety', 'depression', 'formal diagnoses, 'mood state', 'emotion', 'happiness', 'self-esteem', 'diagnosis', 'questionnaire', 'loneliness', 'PHQ-9', 'PHQ-2', 'PC-PTSD', 'GAD-7', 'GAD-2', 'HDRS', 'EQ-5D', 'SF-36', 'GHQ'
Secondary health outcome (physical health): 'physical health', 'BMI', 'waist-circumference ratio', 'cardiovascular health', 'blood pressure', 'heart rate', 'cholesterol', 'cortisol', 'pulse rate', 'MRI results', 'physiological health'
Socio-economic determinants of NOEs use: 'age', 'income', 'sex', 'ethnicity', 'social-economic

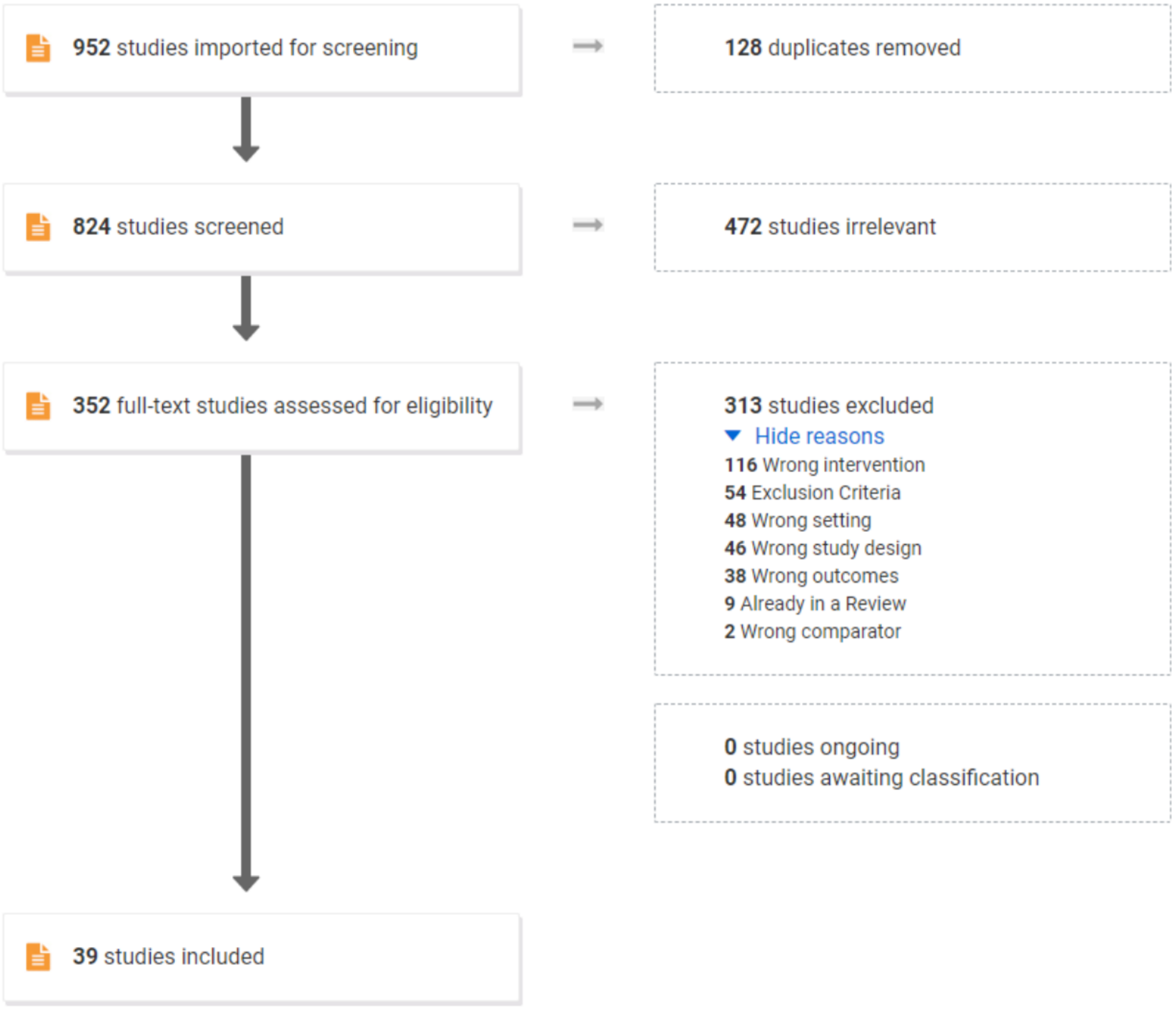

952 studies imported for screening
128 duplicates removed
824 studies screened
472 studies irrelevant
352 full-text studies assessed for eligibility
313 studies excluded
Hide reasons
116 Wrong intervention
54 Exclusion Criteria
48 Wrong setting
46 Wrong study design
38 Wrong outcomes
9 Already in a Review
2 Wrong comparator
0 studies ongoing
0 studies awaiting classification
39 studies included

Environmental processes

- Environment type (i.e. natural environment vs urban)
- Nature connectedness
- Sensory experiences (i.e. sound, smells, etc.)
- Biodiveristy and presence of animals
- Pollution (i.e. air and heat related)

Social processes

- Presence of others
- Social interactions
- Interpersonal processes

Individual processes

- Safety concerns, fear, stigma, social predjudice
- Socio-demographics (i.e. SES, deprivation, age)
- Individual changes (i.e. changing identities, frienships, etc.)

Structural processes

- NBI design and quality (i.e. group organisation, transportation, staff knowledge, time and material resources, duration, activity)
- NOE design and quality (i.e. presence of micro-features of the environment)
- Accessibility (e.g. distance, proximity, pricing, transportation)

PA opportunities

- PA activity type (e.g. swimming, walking, sailing, running, etc.)
- Engagement with nature itself (use vs exposure)

Stress reduction opportunities

- Stressful life events

Study design and quality

- Study design (e.g. participant recruitment, etc.)
- Study quality (e.g. cross-sectional vs RCT, etc.)

Concepts	NCBI search strategy keywords
Green care	Search: (((((((((((((((Green care) OR (green therapy)) OR (nature-based interventions)) OR (nature therapy)) OR (outdoor therapy)) OR (green spaces)) OR (green skills)) OR (green social care)) OR (nature-based therapy)) OR (green prescribing)) OR (green prescriptions)) OR (social prescribing)) OR (green exercise)) OR (green interventions)) OR (green health)) OR (ecotherapy)
Blue care	Search: (((((((((((blue care) OR (blue spaces)) OR (blue interventions)) OR (nature-based interventions)) OR (social prescribing)) OR (blue prescriptions)) OR (blue prescribing)) OR (blue therapy)) OR (blue skills)) OR (blue activities)) OR (blue exercise)) OR (blue interventions)
Mental Health	Search: ((((((((((((((((((((((((mental health) OR (EQ-5D)) OR (wellbeing)) OR (life-satisfaction)) OR (happiness)) OR (emotion)) OR (mood state)) OR (stress)) OR (anxiety)) OR (depression)) OR (GAD-2)) OR (GAD-7)) OR (PC-PTSD-5)) OR (PHQ-2)) OR (PHQ-9)) OR (HDRS)) OR (SF-36)) OR (GHQ)) OR (questionnaire)) OR (formal diagnosis)) OR (diagnosis)) OR (self-esteem)) OR (loneliness)
Physical Health	Search: ((((((((((physical health) OR (physiological health)) OR (bmi)) OR (waist-circumference ratio)) OR (cardiovascular health)) OR (blood pressure)) OR (heart rate)) OR (MRI results)) OR (pulse rate)) OR (cholesterol)) OR (cortisol)
Social Determinants of Health	Search: (((((((((age) OR (income)) OR (sex)) OR (ethnicity)) OR (socio-economic status)) OR (social gradient)) OR (deprivation)) OR (geography)) OR (employment)) OR (social determinants)
Environmental Determinants of green and blue spaces utilisation	Search: (((((((((((((biodiversity) OR (access)) OR (accessibility)) OR (quality)) OR (safety)) OR (crime)) OR (infrastructure)) OR (public transport)) OR (individual will)) OR (motivation)) OR (pollution)) OR (temperature)) OR (weather)) OR (distance)
Full Search History	Search: (((((((((((((((((biodiversity) OR (access)) OR (accessibility)) OR (quality)) OR (safety)) OR (crime)) OR (infrastructure)) OR (public transport)) OR (individual will)) OR (motivation)) OR (pollution)) OR (temperature)) OR (weather)) OR (distance)) AND ((((((((((age) OR (income)) OR (sex)) OR (ethnicity)) OR (socio-economic status)) OR (social gradient)) OR (deprivation)) OR (geography)) OR (employment)) OR (social determinants))) AND (((((((((((physical health) OR (physiological health)) OR (bmi)) OR (waist-circumference ratio)) OR (cardiovascular health)) OR (blood pressure)) OR (heart rate)) OR (MRI results)) OR (pulse rate)) OR (cholesterol)) OR (cortisol))) AND (((((((((((((((((((((((mental health) OR (EQ-5D)) OR (wellbeing)) OR (life-satisfaction)) OR (happiness)) OR (emotion)) OR (mood state)) OR (stress)) OR

	(anxiety)) OR (depression)) OR (GAD-2)) OR (GAD-7)) OR (PC-PTSD)) OR (PHQ-2)) OR (PHQ-9)) OR (HDRS)) OR (SF-36)) OR (GHQ)) OR (questionnaire)) OR (formal diagnosis)) OR (diagnosis)) OR (self-esteem)) OR (loneliness))) AND (((((((((((blue care) OR (blue spaces)) OR (blue interventions)) OR (nature-based interventions)) OR (social prescribing)) OR (blue prescriptions)) OR (blue prescribing)) OR (blue therapy)) OR (blue skills)) OR (blue activities)) OR (blue exercise)) OR (blue interventions))) AND (((((((((((((((Green care) OR (green therapy)) OR (nature-based interventions)) OR (nature therapy)) OR (outdoor therapy)) OR (green spaces)) OR (green skills)) OR (green social care)) OR (nature-based therapy)) OR (green prescribing)) OR (green prescriptions)) OR (social prescribing)) OR (green exercise)) OR (green interventions)) OR (green health)) OR (ecotherapy)) Filters: from 1980 - 2020

Authors and Date	*Country*	*Study Design*	*Population*	*Natural environment(s)*	*Intervention or exposure*	*Key findings*	*Barriers (B) or Facilitators (F)*

#	Authors and Date	Country	Study Design	Population	Natural environment(s)	Intervention or exposure	Key findings	Barriers (B) or Facilitators (F)
1	*Sprague N., Berrigan D., and Ekenga C. (2020)*	USA	Interventional study (non-randomized experiment) – with mixed-methods	**Children** (n=122; age range: 10-15-year-old)	Green spaces – urban forest parks, camping trips, urban farms, cave trips	NBE - Nature-Based Education	- Statistically significant positive changes in **STEM** capacity (+44%) and **HRQoL** (+46%) for participating students.<br>- Qualitative data highlighted the intervention's **educational** and **health** benefits.	- **Age** (F) - the older the child, the more active they will be, and the more benefits they will have on **HRQoL**. No effect for STEM.<br>- **Duration** - the longer tended to have more beneficial effects on **STEM capacity**.<br>- **Stressful life events** - the more one experiences, the less likely they will have benefits from NOEs.
2	*Arnberger A., Eder R., Allex B., Ebenberger M., Hutter H.P., Wallner P., Bauer N. , Zaller J. and Frank T.(2018)*	Austria & Switzerland	Interventional study (field experiment) – using mixed-methods	Adults (workers and university students) (n=22, age mean=26.7; SD=4.1)	Both - one urban city, two meadows (managed vs unmanaged) and river in mountain area	Wilderness expedition – walking and interacting (i.e. viewing) nature	- While differences measured on the physiological level between urban built and natural sites were **marginal** (on DBP not SBP), psychological measures showed **higher health benefits** of the **natural environments**	- **Environment type** (B/F) - river and alpine mountain meadow had the highest health benefits in terms of restoration, BP, and perception of beauty; but all sites recorded an improvement on calming and positive effect post-intervention.

				61 and M=40; age range: 50–80 years old).	Area (formal land cultivated))		- However, **no statistically significant differences** in improvement were found between the intervention and control groups for any of the outcome measures.<br>- **Fatigue decreased** to a value below the suggested cut-off for mental fatigue (< 10.5) in the intervention group, but not in the control group.	
13	*Pratiwi P.I., Xiang Q. and Furuya K. (2019)*	Japan	Interventional study (field experiment) – using quantitative methods	Adults and older adults (local residents) (n=12 in spring; F=6 and M=6; mean age: 66.4) and (n=12 in summer; F=7	Green spaces – across three sites: urban city site and two viewing spots in urban park	Viewing cherry blossom trees and fresh greenery in urban parks VS urban city in Spring and August	- **Viewing cherry blossoms and fresh greenery** in urban parks led **to lower blood pressure** in spring and early summer than viewing city areas in spring and early summer.<br><br>- The results of this study	- **Seasons** (F) - positive mood states were higher in spring as well as lowered mood disturbances; whereas state-anxiety levels were lower in early summer.<br>- **Environment type** (F) - green spaces > urban city for physiological and

				and M=5; mean age: 65.75)			suggest that viewing urban parks results in physiological and psychological relaxation.	psychological effects of NBI.<br>- **Biodiversity and surrounding features** (B/F) (i.e. mosquitoes, sun, temperature, etc.) - thought to have increased heart rate and modified BP measurements, when viewing urban parks in both seasons.<br>- **Presence of water** (F) - associated with a significant **positive effect** and high perceived restorativeness.<br>- **Traffic** (B) (i.e. people or noises from vehicles) - could be responsible for altering BP measures in urban city.

14	*Byström K., Grahn P. and Hägerhäll C. (2019)*	Sweden	Interventional study (field experiment) – using qualitative methods	Children (with disabilities, i.e. autism)<br>(n=9; mental age range: 4–6 years)	Both – immersion in a farm and surrounding nature (no specifications)	**KOMSI treatment** – nature therapy for treating children with disabilities (i.e. horseback riding, free play, etc.)	- The intervention led researchers to conclude on three key benefits of the intervention: 1) reduce stress and instill calm, 2) arouse curiosity and interest, and 3) attract attention spontaneously.<br>- These three perceived benefits are related to **vitality forms**. It is argued that the **vitality forms** from nature and animals are favorable for effecting development-promoting interactions with a therapist.	- **Therapeutic environment (F/B)** - if in nature it can trigger positive or negative responses for the child (i.e. not all autistic children would appreciate being out)<br>- **Presence of animals** (F) - for this subgroup, animals and nature facilitated communication and alleviated stress.
15	*Ana BY, Wanga D., Liua XJ., Guanb HM., Wei HX. and Renb ZB. (2019)*	China	Interventional study (field experiment) – using quantitative methods	Adults (undergraduates in horticulture)	Green spaces – across three types of forests: 1) Maple,<br>2) Birch<br>3) Oak	Forest Bathing in three types of forests	- This study looked at the relationship between environmental factors (temperature, RH, light intensity, and light spectrum)	- **Tree species** (F) - maple> oak> birch for HR improvements. Yet, birch forests still had HR improvements, and was the only one to demonstrate that at

				(n=13; M=7 and F=6; mean age: 21 years old)			and physiological changes (SP, DP, and HR).<br>- Pre--forest-bathing **temperature** and **spectrum** can impact the response of blood pressure due to the "law of the initial value".<br>- HR was influenced positively by visits to maple >oak>birch trees.<br>- Authors recommend visitors to walk in **maple** forests to obtain cardiovascular and autonomic nervous system well-being.	lower levels of BP to begin with.<br>- **Temperature** (B) - Pre-forest bathing temperature can negatively impact the response of BP if PPTs felt too cool and moist.<br>- **Light spectrum** (B) - pre-forest bathing spectrum can negatively impact the response of BP if high G/B ratio are too extreme.
16	*Leavell M.A., Leiferman J. A., Gascon M., Braddick F., Gonzalez J. C. and Litt J. S. (2019)*	USA	Literature review	Across age groups	Both – across several types of natural environments	Several types of NBIs being reviewed here - i.e. water rafting, horticulture, green exercise	- Nature-based social prescription increases **social connectedness** and influences **physical health** and **mental well-being** by certain	- **Intrapersonal processes** (F) - give way to social connections and longer-term health outcomes.<br>- **Interpersonal processes** (F) - improves social connections and

							intrapersonal, interpersonal, and environmental pathways.<br>- NBI practice represents a low cost, creative intervention to **strengthen social networks, reduce stress, and facilitate social connectedness** among participants and providers.	health outcomes by promoting social involvement, relatedness, and shared learning.<br>- **Environmental processes** (B/F) – such as access to nature, perceived neighbourhood attachment, and perceived aesthetics.
17	*Hunter R.F., Cleland C., Cleary A., Droomers M., Wheeler B.W., Sinnette D., Nieuwenhuijsen M.J. and Braubachi M. (2019)*	USA, Australia, UK	Meta-narrative<br><br>evidence synthesis	Across age groups	Urban green spaces – i.e. urban parks, rooftops, parking lots, etc.	Any NBI intervention that has only physical changes to the UGS or with health promotion to tackle inequalities	- There was strong evidence for: 1) **park-based and greenway/ trail interventions** employing a **dual** approach (i.e. a physical change to the UGS and promotion/ marketing programmes); 2) **Greening of vacant lots** which reduced stress and social benefits (e.g.	- **Changes to the built environment in parks** (with dual-approach) (F) - provision of signage and community garden, improvements in existing playing fields, replacement of old playground equipment, installation of outdoor gyms, improved footpaths and clearing of rubbish and vandalism all increased

							reduction in crime, increased perceptions of safety); 3) **Greening of urban streets** and SuDS for managing storm water had environmental benefits as well.	individual's park use, physical activity and the latter two improved QoL and perception of safety.<br>- **Proximity** to newly developed walking/ cycling routes (F) - increased use of these UGS.<br>- **Greening of vacant lots** (F) – reduced perception of unsafe environment and bolster use of these UGS.
18	*van den Bosch M. and Sang O. (2017)*	Not provided	Systematic review (of reviews)	Across age groups	Both - (i.e. green infrastructure, biodiversity, blue environments, etc.)	Interventions in urban natural environments	- There is strong evidence on the effect of urban nature **on affect state**.<br>- There is strong evidence on the effect of urban nature on **urban heat reduction**.<br>- **Positive affect and heat reduction** can mediate urban nature's effect on **mortality**.	- **Micro-features** (F)<br>- **Conditions of natural environments** (B/F)<br>- **Perceived quality** (F)<br>- **Accessibility** (B/F)<br>- **Safety** (B)

19	*Houlden V., Weich S., de Albuquerque J.P., Jarvis S. and Rees K (2018)*	Europe USA Canada, Australia	Systematic review	Adolescents, adults, and older adults	Green spaces – mixed definition that encompasses vegetated areas and/or wilderness	Not interventions per se, but includes studies with walking in GS as measure for visits to GS	- There was **adequate evidence** for associations between the **amount of local-area greenspace and life satisfaction** (hedonic wellbeing), but **not personal flourishing** (eudaimonic wellbeing).<br>- Evidence for associations between **mental wellbeing and visits to greenspace, accessibility, and types of greenspace** was limited.<br>- There **was inadequate evidence** for associations with views of greenspace and connectedness to nature.	- **Views of greenspace** (B/F) - if looking at unpleasant urban/rural views it will have negative association with mental health.<br>- **Connection with nature** (F) - the more connected one is with nature, the more health benefits (i.e. life satisfaction, happiness, affect, QoL) one will experience. This is modulated by **being actively engage** in nature, however.<br>- **Visits to greenspaces** (F) - active immersion in wilderness was found to lead to greater happiness, affect and attention.

20	*McCormick R. (2017)*	USA, Spain and others not specified	Systematic review	Children (age range: 0-18)	Green spaces – wooded playgrounds, natural habitats, gardens, etc.	Not interventions per se but does include studies who used walking in NOE as measure for visits to green space.	- **Access** to green space is important to the **mental well-being**, **overall health,** and **cognitive development** of children. It promotes **attention restoration**, moderates the impacts of **stress**, improves **behaviours** and **symptoms of ADHD** and was even associated with **higher standardized test scores**.	- **Proximity to GS** (F) - only passive exposure but the closer one lives to nature, the better health outcomes they have.<br>- **Physical activity** in NOE (F) - **walking** in nature for children vs in urban environment led to improved attention and spatial working memory - which can help children with ADHD focus better.
21	*Barrett. J., Evans S. and Mapes N. (2019)*	UK	Literature review	Older adults (residents of dementia care settings)	Green space – garden areas within dementia care settings	Green dementia care - includes many type of NBIs (i.e. horticulture, walking, gardening, etc.)	- Compelling evidence for **several health and wellbeing benefits** associated with green dementia care (i.e. improved wellbeing, social interactions, stress-reduction and restorative effects, self-	- **Safety concerns** (i.e. fear of falling in garden) (B)<br>- **Staff attitudes** and **lack of staff education** and awareness (B)<br>- **Social prejudice and stigma** (B)<br>- **Limited staff** to accompany residents and **limited resources (B)**<br>- **Weather** (B)

							worth and confidence.)<br>- Evidence base is stronger regarding the **barriers and facilitators** to accessing nature for this population - **staff education and care culture** is critical to the success and effective use of the garden for such residents.<br>- **Design** of the outdoor space need to ensure that these spaces are **visually and physically accessible** for its residents.	- **Self-perception of being too old** and **lack of confidence** (B)<br>- Poor physical and visual access<br>- **Poor garden design** (B) (i.e. lack of resting places and weather protection)<br>- **Care culture NOT person-centred** (B)
22	*Ottoni C.A., Sims-Gould J., Winters M., Heijnen M. and McKay H.A. (2016)*	Canada	Observational study (participant observation) – using	Older adults (60+) (n=28; F=17 and M=11, age range: 61-89, in 2012; n	Both areas – three areas in parks in Vancouver with features of GS and BS	Physical activity in nature - recorded as step counts/day (mean)	- **Neighbourhood environments** influence health and well-being as people age.<br>- There are strong interconnections between **built**	- **Amenities** (F) – i.e. benches, seen as a necessity to promote social interactions and positive experiences.<br>- **Ability to engage in other type of activities** (F) – i.e.

			qualitative methods	=22, F=12 and M= 10; in 2014)			**and social environments**.<br>- **Microscale features** can enable older adults' to accommodate to their abilities.<br>- **Benches** can promote mobility and social connectedness for older adults.<br>- **Microscale features**, like benches, are a prudent investment for communities.	family or friends activities, going to the pub, going to the gym featured more prominently than benches in relation to their mobility.<br>- **Injury** (B) - to use the outdoor environments.<br>- **Wildlife** (F) - promoted feelings of enjoyment and calmness. Also helped in creating routines/familiarity with these spaces.<br>- **Presence of other people** (B/F) - a negative experience for older adults if too many people use benches. But seeing people around them also provided positive feelings opportunities for social interactions.<br>- **SES** (B) - **accessibility and availability** of GS and BS was more common for older adults with higher

							compared to the built one.	
3	*Gargiulo I., Benages-Albert M., Garcia X. & Vall-Casas P. (2020)*	Spain	Observational study (exploratory fieldwork) – using qualitative methods	Adults stream users (N=30; F=14 and M=16, age range: 27-65+ years old)	Green – urban stream corridor	Leisure-Time Physical Activity (LTPA)	- Social and physical factors of the environment are perceived as either barriers or facilitators, with different nuances and importance, depending **on each type of user**.<br>- Also, for the same type of user, factors perception also depends on gender; whereby safety was important for women engaging in LTPA.	- **Safety** (B) – women reported lower level of use of blue/green spaces during LTPA if safety was a concern.<br>- **Environmental design** (B/F) - itineraries with **enhanced visibility,** higher **attendance**, pruning of dense vegetation and provisioning of **assistance** in case of need, all promoted engagement in LTPA.<br>- **Accessibility** (F) - stream accessibility and proximity to environments is conductive for LTPA.<br>- **Presence of others** (B/F) - having someone to share the experience with facilitated engagement. But

								social and economic status.
23	*Howarth M., Rogers M., Withnell N. and McQuarrie C. (2018)*	UK	Observational study (cross-sectional) – using mixed-methods	Adults and older adults (suffering from mental disorders (n=47; age range: 35-68 years and average age: 53.2 years)	Green space – garden area created by social enterprise	Therapeutic horticulture	- Quantitative findings showed that participants were working towards **self-reliance**. Qualitative data found similar results.<br>- **Mental health recovery programme** enabled participant **integration** into the community through providing a space to **grow and build self-confidence** while reengaging with society.<br>- The results suggest that using therapeutic horticulture as	- **Positive staff attitudes** (F) - welcoming and non-judgmental attitudes promoted wellbeing and social connection for this population. It also helped people feel safer.<br>- **Activities as a new purpose** (F) – engaging in nature itself improved wellbeing and allowed people to feel a sense of purpose. By developing new skills people felt more confident in their own self and their employability.<br>- **Presence of others** (F) - improved sense of purpose and recovery, as everybody shared

							an intervention within the mental health recovery programme can **support people with mental health problems** to re-engage socially.	same/similar experiences; this helped them move beyond their diagnosis. It also provided opportunities to re-engage with society.
24	*Kabisch N., Matilda van den Bosch M. and Lafortezzae R. (2017)*	U.S., Germany France Spain Denmark, Bulgaria, Austria, Sweden, UK, Japan, Canada and China	Systematic Review	Children and the elderly	Both – features of both GS and BS	Some studies included interventions / active engagement with nature	- There is a tendency for a positive association between urban green and blue spaces and reduced risk factors related to urbanization for children and the elderly as well as the promotion of health-related behaviours and subsequent positive health outcomes.<br>- But the evidence is weak and the results are somewhat inconsistent.	- **Socioeconomic factors** (B) (i.e. deprivation, income, educational level, unemployment) - the lower one's household, the worst their health outcomes, and the lower the relationship between health and nature.<br>- **Air pollution** (B) - act as mediator of the relationship between nature and health - but not if elderly engage actively in NOE (i.e. gardening).<br>- **Heat-related pollution** (B) - the higher the heat in parks, the less use and the worst health outcomes,

								specifically for the elderly.<br>- **Proximity/distance** (F/B) - proximity can modify effectiveness of NBIs.
25	*Shin J.C., Parab K.V., An R. and Grigsby-Toussaint D.S. (2020)*	USA, Australia, Canada, Spain, UK, Netherlands, Lithuania	Systematic review	Across age groups	Green spaces – neighbourhood greenness, visits to GS, engagement in activities related to GS	Several interventions included: walking, gardening, work environment	- **Green space exposure** (through active engagement) is associated with **better sleep quality and quantity.**<br>- Authors suggest **green exercise** and **therapeutic gardening** for future interventions.	- **Time of day for activity** (F) – afternoon walking > morning walks.<br>- **Type of environment (F)** - outdoor > indoor interventions on sleep latency.<br>- **Behavioural preferences** (F) - people had better sleep on weekdays with lower exposure to green spaces. Also preferred vaster expenses of greenspace on weekends and for longer than during weekdays.
26	*Lakhani A., Norwood M., Watling D.P., Zeeman H. and Kendall E. (2019)*	USA, Norway, Netherlands, Australia, Korea, Japan	Systematic review	Adults and older adults (suffering from neurological disability:	Both – includes studies with features from both green and blue environments	Several interventions included: gardening, green care farming, wilderness therapy,	- Given the limited research to date, and the diversity of nature specific activities, it is **not possible to establish**	- **Environment type** (F) - for care farming, evidence is mixed on social health.<br>- **Garden design** (F) - gardens often well arranged, walled

				dementia, stroke,<br><br>and brain injury)		forest therapy	**definitive conclusions** around the efficacy of engaging with nature specific activities on the **psychosocial health** of people with neurological disability.<br>- At best, findings clarify that **engaging** with natural environments contribute to favourable **emotional health outcomes and social health outcomes** for people with dementia.	and preferably in connection with a shielded dementia care unit, may also improve agitation among people with dementia.<br>- **Mobility** (B) - impact of wander gardens on agitation reduction was lower if PPT had ambulatory issues.<br>- **Presence of caregiver** (F) - brought positive emotional health outcomes in patients with dementia, when entering garden.<br>- **Short-term plants** (F) –associated with improved social health vs long-term plants. Possibly due to faster harvesting capacity.<br>- **Active engagement** (F) - psychological health has been favourably impacted only when activities in nature

								involve active engagement.
27	*Kondo M.C., Fluehr J.M., McKeon T. and Charles C. Branas C.C. (2018)*	USA, UK, Netherlands, Canada, Lithuania, Denmark, Germany, Finland, Japan, Italy, Spain	Systematic review	Across age groups	Green spaces – natural environment	Several types of interventions including: viewing nature, walking, exercising, gardening	- This review of experimental, quasi-experimental, and longitudinal studies found **evidence of a positive association** between urban green space and **attention**, **mood**, and **physical activity**, and **negative association with mortality, short-term cardiovascular markers (heart rate), and violence**.<br>- In most cases, it is not possible to observe patterns of findings of association between urban green space exposure and health outcomes (i.e. birth outcomes,	- **Environment type** (F) - natural environment > urban built environment for attention, general health, cardiovascular outcomes (i.e. HR, HRV), mood and emotions (i.e. specifically urban woodlands for restoration).<br>- Biodiversity (F) - found to improve **mood** and **emotions** but is mediated by length of park visit and perceived restoration.<br>- Physical activity (F) - engagement in PA in nature was positively associated with health outcomes in experimental studies VS observational studies.

							stress, BP, cancer, diabetes, etc.).	
28	*Callaghan A., McCombe G., Harrold A., McMeel C., Mills G., Moore-Cherryb N. and Cullen W. (2020)*	Australia, USA, UK, Bulgaria, Belgium, Denmark, Netherlands, Serbia	Scoping review	Across age groups	Green spaces – urban parks, neighbourhood greenness	Several interventions included: horticultural therapy, walking, viewing from indoors	- The majority of studies found a **positive association** between GS and mental health.<br>- Policies to increase urban green space may have **sustainable public health benefits**.	- **Ethnicity** (B) - South Asian children living in more deprived areas and with lower access and quality of GS had more behavioural difficulties VS white British children.<br>- **Deprivation** (B) - quality and access to greenspaces is lower in deprived and lower-income communities,

								leading to worse health outcomes.
29	*Koselka E.P.D., Weidner L.C., Minasov A., Berman M.G., Leonard W.R., Santoso M.V., de Brito J.N., Pope Z.C., Pereira M.A. and Horton T.H. (2019)*	USA	Interventional study (pilot study) – using quantitative methods	Adults (undergraduates, graduates and employees) (n=37; 18–35 years; age mean=22.9 and SD=4.6)	Green	Walking in nature across 3 settings: forest; along roadside, activities of daily living	- This study has found that **moderate-intensity walking** in a forested environment had a **positive impact on psychological health**.<br>- This suggests that completing **physical activity** in greenspaces **amplifies beneficial acute psychological responses** and yields greater improvements in **mental health** than does activity completed indoors or in a built urban environment.	- **Type of environment** (F) - forest walking >roadside> daily activities for positive/negative affect, perceived stress, and anxiety.<br>- **Physical activity** (F) - walking in general brought improved mental health but amplified in forest environment.
30	*Zufferey J. (2016)*	Japan, Australia, China, USA, New			Both – GS and BS elements (not specific)	Not specified per se, but includes studies with walking/	- This literature review shows moderate to strong empirical evidence for the	- **Age** (F) - children and young adults seemed to benefit more from exposure to GS and

		Zealand, Canada	Systematic review	Across age groups		exercise in GS and BS	positive influence of **contact** with green and blue spaces and **mental and physical health** and **low evidence** for influences on **social cohesion**.<br>- It also shows that health impacts may vary according to the **population group** considered (e.g. children, people with low socio-economic status who benefit more from exposure to these environments).	BS, especially via physical activity; which had combined effects on physical and mental health.<br>- **SES** (B/F) - lower SES households tended to have more health benefits associated with exposure to green and blue spaces.<br>- **Type of environment** (F) - natural environments > urban built on emotional wellbeing.
31	*Costello L., McDermott M-L., Patela P. and Dare J. (2019)*	Australia			Blue – the ocean and surrounding beaches	Ocean swimming	- All the ocean swimming groups studied were united by their **routine** of beach swimming, by their **love of the ocean**, and their conviction that	- **Type of environment** (F) - swimming in the ocean VS public/private pool.<br>- **Biodiversity** (B/F) - when seeing fishes, dolphins, whales, etc. people experienced

			Observational study (ethnography) – using qualitative methods	Older adults (self-organised ocean swimmers) (n=10; F=7 and M=10; age range: 55-80+ years)			their ocean swimming practice **as part of a group** was beneficial for their social connectedness, wellbeing and physical and mental health.	positive emotions and pleasurable experiences. However fear of sharks led to negative emotions, despite increasing social cohesion.<br>- **Type of activity** (F) - swimming > other type of outdoor exercise, as it was low-impact. It would also help in alleviating stress.<br>- **Weather** (F) - despite cold and rainy weather, PPTs would still engage in swimming, as their commitment to the group was the priority.<br>- **Group membership** (F) - PPTs recognised that they would not derive the same enjoyment, pleasure and health benefits w/o group.
32	*Birch J., Rishbeth C. and Payne S.R. (2020)*	UK		Adolescents and adults (n=24;	Both – urban parks in Sheffield (UK)	Arts workshop and interviews in nature	- Deteriorating landscapes, young people's shifting identities and perceived time pressures	- **Poor quality of urban environment** (B) – or urban deprivation, was more impotant than

			Observational study (case study) – using qualitative methods	F=14 and M=10; age range: 17-27 years, with n=9 experiencing mental difficulties and n=15 living in an area of urban deprivation).			disrupted support.<br>- Overall young people expressed how urban nature encounters were experienced **as accepting and relational**, offering a **stronger sense of self**; feelings of escape connection and care with the human and non-human world.	ethnicity and SES across PPTs.<br>- **Presence of others** (F/B) - having someone with you during a visit to an urban environment was positively experienced (i.e. wanting someone to share experience with), or negatively experienced (i.e. wanting to be alone).<br>- **Individual factors** (B/F) - pressures, changing priorities and changing friendships all have their mediating role.
33	*Wood E., Harsant A., Dallimer M., ronin de Chavez A,, McEachan R.R.C. and Christopher Hassall (2018)*	UK	Observational study (cross-sectional) – using quantitative methods	Adults and older adults (users of local parks in deprived areas) (n=128; F=59 and M=69; age range: 18-76+ years)	Green	Visits to greenspaces - survey conducted at the park entrance	- Authors found that **biodiversity** and site facilities were positively correlated within urban parks. However, we found that only **biodiversity was related to perceptions of psychological restoration** amongst a multi-	- **Biodiversity** (F) - the more biodiversity a park had; the more people would benefit from psychological restoration.<br>- **Amenities** - positively correlated with urban parks, but no effects on psychological restoration.

								can be a barrier when perceived as **safety risk** (i.e. walkers vs runners/cyclists and vice-versa).<br>- **Environment type** (F)
4	*Denton H. and Aranda K. (2019)*	UK	Observational study (ethnography) – using qualitative methods	Adults (regular swimmers and existing club members) (n=6; F=3 and M=6, age range: 38-73 years old)	Blue space - sea swimming club (Brighton, UK)	Sea swimming	- The swimmers found sea swimming **transformative**, (resulting in changes in the swimmer's experience of themselves); **connecting (**experiencing a sense of connection to nature, place and others); and **re-orientating** (as swimmers seemed to use this disruption to reconnect to what they consider is important), through **the disruption to the sense of time, space and body**, swimmers find alternative and	- **Physical activity** - engaging actively in the sea by swimming was critical to gaining health benefits (i.e. emotional and physical health)<br>- **Fear and stigma** - negative body image can impact one's engagement in sea swimming; but also fear of the challenges from the sea.

							ethnic group of PPTs.<br>- These findings suggest that urban planners should aim to enhance **ecological diversity** in urban green spaces.	- **Ethnicity** - no effects found.
34	*Corazon S.S., Sidenius U., Poulsen D.V. , Gramkow M.C. and Stigsdotter U.K. (2019)*	Europe, Asia, Australia	Systematic Review (without meta-analysis)	Adults and older adults<br>.	Both – all types of outdoors natural green environments	All types of sedentary and light exercise activities, in all time durations in nature	- The synthesis of the results points towards outdoor, nature-based exposure having a positive effect on different **emotional parameters**, related to stress relief. The studies into physiological measures showed more equivocal results.<br>- The general use of **self-referred individuals** imposes a potential strong bias.	- **Type of environments** (F) – natural environments vs control had positive association with emotional outcomes (i.e. positive affect, perceived stress and wellbeing/QoL), and negative association with negative affect. This could not be found for physiological measures – (too heterogeneous).

35	*Maund Pgreen.R., Irvine K.N., Reeves J., Strong E., Cromie R., Dallimer M. and Davies Z.G. (2019)*	UK	Interventional study (pilot study) – using mixed-methods	Adults and older adults (already registered with the community mental wellbeing service and diagnosed with depression and/or anxiety) (n=16; F=8 and M=8)	Blue – wetlands	**Wetland NBI** – guided walking, bird watching or other activities (i.e. canoeing) done in nature over a six-week period.	- There were significant improvements in **mental health** across a range of indicators, including **mental wellbeing, anxiety, stress and emotional wellbeing**. Participants and healthcare professionals cited additional outcomes including **improved physical health** and **reduced social isolation**.<br>- The wetland site provided a **sense of escape** from participants' everyday environments, facilitating relaxation and **reductions in stress**.<br>- Wetland **staff knowledge** of the natural world,	- **NBI design** (F) - to be successful, NBIs need to take into account transportation, staff knowledge and group dynamics.<br>- **Biodiversity** (F) - the presence of water, diverse wildlife and the inherent peacefulness of wetlands were positively experienced with the intervention.<br>- **Session content** (B/F) - most PPTs preferred if there was only ONE activity vs many → less stress and anxiety that way.

							**transportation** and **group organisation** also played a role in the intervention's success.	
36	*Benjamin-Neelon S.E., Platt A., Bacardi-Gascon M., Armstrong S., Neelon B., Jimenez-Cruz A. (2019)*	Mexico	Observational study (cross-sectional) – using quantitative methods	Children (n=102, age rage: 3-5 years; in Ensenada (M=29 and F=21) and in Tijuana (F=27 and M=25)	Green spaces – urban parks in two cities (Tijuana and Ensenada)	Time spent in GS (measured with GPS)	- **Greater time** in greenspace was associated with **decreased sedentary time**.<br>- **Greater time** in greenspace was associated with **increased physical activity**.<br>- Associations were mainly driven by children in **Tijuana** compared to Ensenada.<br>- Time spent in greenspace was **not associated** with body mass index **(BMI) z-score.**	- **Duration** (F) - the greater the time spent in greenspace, the less sedentary time these children will experience, but also the greater their MVPA will be (physical activity).
37	*Coventry P.A., Neale C. Dyke A., Pateman R.*	UK		Adults and older adults (conservation volunteers) (n=45; F=20	Green spaces – across three sites: Askam Bog, St Nicks natural reserve and a large green field with	Three interventions: group walking, conservation	- Undertaking **purposeful activity** in public green space has the potential to **promote health** and prevent	- **Location** (F)- effects differed for **stress** across locations, meaning that the location of the GS, over the type of activity, was

	and Cinderby S. (2019)		Interventional study (field experiment) – using mixed-methods	and M=25; age mean: 43.8)	surrounding woodland, adjacent to a semi-urban housing development	, citizen science	**mental ill health**. Undertaking such activities in locations where people have the **most connection** might confer additional benefits.<br>- **Social interaction, physical activity and restoration** were all implicated as potential mechanisms by which activities in public green spaces might lead to improved mental health.	an important factor in reducing stress - which was explained by an enhanced **place attachment** and **place identity** at this location.<br>- **Type of activity** (F) - although not shown quantitatively, **conservation and citizen science** were both associated with deeper **sense of purpose** by providing **learning opportunities**, and because it conferred **co-benefits** to health, wellbeing and to nature itself.
38	*Britton E., Kindermann G., Domegan C. and Carlin C. (2018)*	Europe, USA, Canada, New Zealand, Israel	Systematic review	Across all age groups – but with pre-existing condition	Blue space – wilderness, sea, urban/ semi-urban areas (beach, city), or mix of these	Several interventions included in BS: surfing, Dragon Boat Racing (DBR), sailing fly fishing kayaking, canoeing, at the beach, swimming,	- Blue care have **direct benefit** mental health and psycho-social wellbeing.<br>- There was also evidence for **greater social connectedness** during and after interventions, but results were **inconsistent**	- **Access** (F/B)<br>- **Lack of resources/ equipment** (B)<br>- **Fears/stigma** (B) - associated with personal abilities, level of fitness, environment, social and cultural norms, diagnosis of illnesses and level of appropriate training for those

						(as part of a kayaking intervention), and scuba diving	and mixed; with very few findings for physical health.<br>- Findings suggest how **activities** in BS, rather than particular **qualities** of BS, might contribute to rehabilitation and health promotion.	delivering intervention.<br>- Gender<br>- **Seasickness** - due to poor weather (B)<br>- **Fatigue/tiredness** - post-intervention (B)
39	*Saadi D., Schnell I., Tirosh E., Basagaña X. and Agay-Shay K. (2020)*	Israel	Interventional study (field experiment) – using quantitative methods	Adults (women specifically) (n=120; age range: 20-35, from two small cities in the north of Israel, of whom n=48 were Arab and n=24 were Jewish women (n= 72)	Green spaces – across Afula-urban park, Afula-city center, Afula-residential area, Nazareth urban park, Nazareth-city center and Nazareth-residential area VS home (control)	Viewing and waling in nature while sitting on benches across 6 different sites	- **Visits to urban parks** compared to staying in the home environment had beneficial short-term changes in **psychological**, **physiological**, and **cognitive** responses, regardless of ethnicity.<br>- The changes could **not be attributed** to the investigated **mediators**.<br>- Women should be encouraged to go outdoors and specifically visit parks to	- **Environment type** (F/B) - Arab woman demonstrated improvement in most outdoor environments, while for the Jewish woman, improvement was reported mainly in parks, but not in any other urban environment.<br>- **Socio-demographic** (ethnic preferences) (F) - whereby benefits were stronger in intra-ethnic parks.<br>- **Comfort level at home** (F/B) - more comfort at home for Jewish women vs

							improve their psychological and physiological health	Arab women, which could have reduced positive effects of outdoor environments considered less comfortable for this subgroup.

							expanded perspectives about themselves and their world.	
5	*Finlay J., Franke T., McKay H. and Sims-Gould J. (2015)*	Canada	Observational study (participant observation) – using qualitative methods	Older<br><br>adults (community dwellers; T1: N=27; T2: N=19,<br><br>age range: 65-86 years old)	Both - urban parks in neighbourhood, with green and blue features	Walking	- Older adults have distinct **therapeutic relationships** with landscapes.<br>- Nature **can promote the physical, mental, and social health** of older adults.<br>- Blue space in particular embodies important therapeutic qualities for older adults.	- **Safety** (B/F)- can be experienced differently by people.<br>- **Accessibility** (B/F) - the least accessible, the worst the experience<br>- **Personal perception** (B/F) - the same place could evoke feelings from enjoyment to indifference to concern due to traffic, park maintenance, walkability, etc.

6	*McEwan K., Richardson M., Sheffield D., Ferguson F.J. and Brindley P. (2019)*	UK	Interventional study (RCT) – quantitative methods	Adults (18+) residing in<br><br>Sheffield and owning a smartphone<br><br>(N=148)	Greenspace – urban park vs control (urban built)	Social prescription app on smartphone design to make people **notice** nature (while being in nature)	- Using a social prescription using a Smartphone app (noticing nature) resulted in statistically significant **improvements** in **wellbeing** for adults in general, and **clinically significant** improvements in wellbeing for those classed as having a mental health difficulty.<br>- These improvements were more pronounced in the **green space** condition, despite improvements still in control.	- **Environment type** (F) – both built and green environment yielded short-term benefits on wellbeing through **nature connectedness**; but only green space condition had sustained effects after one-month follow-up.<br>- **Previous experience with nature** (F) – from childhood or in the last year, both have positive effects on wellbeing.<br>- **Positive affect** (F) – predictor of wellbeing in green condition.
7	*Nicolosi V., Wilson J., Yoshino A.& Viren P. (2020)*	USA	Interventional study (field experiment) – using quantitative methods	Adults (undergraduates)<br><br>(n=63; F=31 and M=32;	Blue spaces – the coast vs control (urban side-road)	Coastal and urban walk	- Significantly higher average **perceived restoration** scores were associated with the natural (coastal) walk.<br>- **Coastal exposure, sound quality**	- **Perceived sound level** (F) - higher perceived sound level was a significant predictor of a restorative experience.<br>- **Environment type** (F) – if in natural environment, then increased

				age mean=20.4)			**and type** were rated as very good and more natural than the sidewalk respectively and were significant predictors of **a restorative experience.**	perceived restoration.
8	*Cheesbrough A.E., Garvin T., Nykiforuk C.I.J. (2019)*	Canada	Observational study (case study)– using qualitative methods	Adults and older adults (residents around one of the five selected NAP); (n=33; F=18 and M=15; age range=29-87 years)	Both – within five Natural Area Parks (blue and green features present)	Nature photography and reflection in nature	- **Proximity** to natural areas facilitated frequent and spontaneous visits.<br>- **Repeat visits** fostered intimacy with the space over time.<br>- Participants felt 'away from the city' while in the middle of the city.<br>- Participants reported **physical, spiritual and psychological therapeutic impacts**.<br>- Natural areas facilitated **connections to nature**, **self**, **companions**, and **others**.	- **Proximity** (F) - increase engagement **in physical activity** and therefore promotes improved health benefits.<br>- **Topography** - more difficult terrain were **motivational** for users of NAPs.<br>- **Sensory qualities** (F) - facilitated a **positive experience** of being in nature, and therefore allowed for this visit to be a **restorative experience**.<br>- **Safety** (B) - main barrier to using these areas (whether for fear of humans or wildlife).

9	*Barton J., Bragg R., Pretty J., Roberts J., and Wood C. (2016)*	South Africa & Scotland	Interventional study (field experiment) – using quantitative methods	Adolescents (n=130; F=74 and M=57; age range: 11-18 years old)	Both – in a game reserve and a loch	Wilderness expedition in natural environment s	- Environment, gender, and the length and location of expeditions significantly contributed to PPTs' changes in self-esteem (SE) and nature connectedness (NC).<br>- PPTs living in urban environments and going to local wilderness for a short duration will receive the same amount of benefits SE and NC as PPTs who live in a **rural location** and are immersed in a remote wilderness for longer.	- **Gender** (F) - males had higher self-esteem at start, but significant increase in SE for females at the end.<br>- **Duration** (F)- even short durations of expeditions can have benefits on nature connectedness and self-esteem.

10	*Lanki T., Siponen T., Ojala A., Korpela K., Pennanen A., Tiittanen P., Tsunetsugu Y., Kagawa T. and Tyrväinen L. (2017)*	Finland	Interventional study (field experiment) – using quantitative methods	Adults (female volunteers<br><br>in Helsinki) (n=36;<br><br>age range: 30-60 years old)	Green space (vs control) - an urban forest, an urban park, and a built-up city centre	Each visit: 15 min of sedentary viewing; and 30min of walking	- Beneficial changes in **cardiovascular physiology** were observed in green environments.<br>- Specifically, **lower blood pressure** (viewing period only), **lower heart rate**, and **higher indices of heart rate variability**.<br>- **Large** urban park and **extensively managed** urban woodland had positive influence, but the overall perceived restorativeness was higher in the **woodland**.<br>- This may be explained by **stress relief** and **lower air pollution** and **noise exposure**.	- **Stress relief** (F) – the more relaxed one is in NOE, the better their health outcomes.<br>- **Air pollution** (B) – the higher the air pollution, the worst the health outcomes.<br>- **Noise exposure** (B) – higher noise engenders higher stress, and therefore lower health benefits.

11	*Marselle M.R., Warber S.L. and Irvine K.N. (2019)*	UK	Observational study – using quantitative methods	Adults (volunteers) (N=1,516; age range: 55 years or older)	Both - natural environment (i.e., natural and semi-natural places, farmland, green corridor, coastal area, urban green space, or any mixture of the above)	Nature group walks	- Neither nature group walking, nor doing this frequently, moderated the effects of stressful life events on mental health.<br>- The positive associations of group walks in nature were at a greater magnitude than the negative associations of stressful life events on depression, positive affect, and mental well-being, suggesting an '**undoing' effect of nature group walks**.	- **Stressful life events (B)** – walking can help un-do stress associated with stressful life events by reducing depression and increasing positive affect and wellbeing.<br>- **Presence of others** (B) - can dampen buffering effect of nature on mental health.<br>- **Physical activity** (F) – mechanism by which individuals gain benefits from nature.
12	*PÁLSDÓTTIR A.M., STIGMAR K., NORRVING B., PETERSSON I.F., ÅSTRÖM M. and PESSAH-RASMUSSEN H. (2020)*	Sweden	Interventional study (RCT) – using quantitative methods	Adults and older adults (stroke survivors) (n=101; F=	Green space - Alnarp Rehabilitation Garden (Nature Area (informal and non-cultivated) and the Cultivation and Gardening	Nature-based rehabilitation (NBR) using horticultural therapy	- The patients with sub-acute stroke were highly **compliant** with the intervention. The participants in both the intervention and control groups **improved**.	- **Weather** (B) - not suitable NBR in bad weather.<br>- **Access** (B) - acted as barrier to participation in NBR for some PPTs due to longer travel time to the garden.

Mental Health Outcomes				
Category	*Outcome*	*Reference*	*Association (with NOE)*	*Modulators (Barrier/Facilitator)*
**Psychological health**	HRQoL – health-related quality of life	[131] [142]	Positive	Stressful life events (B), age (B/F), environment type (F) (131); no difference between intervention vs control (142)
	Quality of Life (QoL)	[147][164]	Positive	Changes to the built environment (improved footpaths and clearing of rubbish and vandalism) (F) (147); Environment type (F) (164)
	Wellbeing	[132][136] [141][146] [149][154] [164][165]	Positive/ Mixed effects	**Positive**: Environment type (F) (132); environment type (F), previous exposure to nature as child and last year (F), positive affect (F) only in green space, nature connectedness (F) in both control/ intervention (136); physical activity (F), presence of others (B), stressful life events (B) (141); Interpersonal processes (F), Environmental processes (B/F – based on access, perceived aesthetics, and neighbourhood attachment) (146); Environment type (F) (164); Transportation (F), Staff knowledge (F), Group organisation (F), Biodiversity (F) (165) Air and heat-related pollution (B), Proximity (F), SES (B/F) (154); **Mixed effects**: study design (B); terminology for GS (B) (149);

**Psychological health**	Hedonic Wellbeing (life satisfaction)	[149]	Positive	Connectedness with nature (F), Active engagement with nature (F) (149)
	Eudaimonic wellbeing (personal flourishing)	[149]	No effect	n/a
	Perceived wellbeing	[134][151] [152][153] [161][168]	Positive	Environment type (F), physical activity (F) (134); Environment type (F), Active engagement with nature (F) Safety concerns (B), Staff attitudes and lack of staff education and awareness (B), Social prejudice and stigma (B), Limited staff and resources (B); weather (B/F); Negative self-perception and lack of confidence (B); Poor physical and visual access (B), Poor garden design (B) (i.e. benches, weather protection), Care culture NOT person-centred (B) (151); Micro-features (i.e. benches) (F) (152); Positive staff attitudes (153); Environment type (ocean > public pools) (F) (161); Access (B/F), Environment type (F), Fear and stigma (B), Lack of resources/ equipment (B) (168)
	Perceived mental health	[135]	Positive	Safety (B), accessibility (B/F), personal perceptions (B/F) (135)
	Depression	[141][142] [156][158]	Negative	Stressful life events (B), physical activity (F) (141); no difference between intervention vs control (142); Presence of caregivers (156);

**Psychological health**				Physical activity (F), Environment type (F) (158)
	Anxiety	[142][143] [156][158] [159][165]	Negative	No differences between intervention vs control (142); Season - Summer (F) (143); Presence of caregivers (156); Physical activity (F), Environment type (F) (158); Environment type (forests>roadside> daily activities) (F), Physical activity (F) (159); Transportation (F), Staff knowledge (F), Group organisation (F), Biodiversity (F) (165)
	Psychological Restoration	[163]	Positive	Biodiversity in urban park (F) (163)
**Social health**	Social isolation	[134][151] [152][153] [161][165]	Negative	Environment type (F); physical activity (F) (134); Environment type (F), Active engagement with nature (F) Safety concerns (B), Staff attitudes and lack of staff education and awareness (B), Social prejudice and stigma (B), Limited staff and resources (B); weather (B/F); Negative self-perception and lack of confidence (B); Poor physical and visual access (B), Poor garden design (B) (i.e. benches, weather protection), Care culture NOT person-centred (B) (151); Micro-features of the environment (benches) (F), Accessibility (B) (152); Positive staff attitudes (F), Presence of others (F) (153); Group membership (F), Environment type (F) (161); Transportation

**Social health**				(F), Staff knowledge (F), Group organisation (F), Biodiversity (F) (165)
	Social connectedness	[144][146] [156][160] [161][162] [168]	Positive	Environment type (F), Presence of animals (F) (144); Intrapersonal processes (F), Interpersonal processes (F), Environmental processes (B/F – based on access, perceived aesthetics, and neighbourhood attachment) (146); Environment type, i.e. farms (F), Harvest speed (F) (156); Lower SES (F), Environment type (F) (160); Group membership (F), Weather (F), Threatening biodiversity (F) (161); Environment type (F), Individual factors (i.e. time pressures, changing identities) (B), Presence of others (B/F) (162); Access (B/F), Fear and stigma (B), Lack of resources/ equipment (B), Environment type (F) (168)
	Social discomfort	[169]	Negative	Environment type (F/B), Ethnicity (B/F) (169)
**Emotional Health**	Positive Affect	[136][142] [148][151] [159][160] [161] [164] [165]	Positive	Environment type (F) (136); Environment type (F), physical activity (F), stressful life events (B) (141); Environment type (forests>roadside> daily activities) (F), Physical activity (F) (159); Environment type (ocean>pool) (F), Biodiversity – if non-threatening (F) (161); Environment type (natural>control) (F) (164) Micro-features (F), Conditions of natural environments (B/F), Perceived quality (F), Accessibility

**Emotional Health**				(B/F), Safety (B) (148); Environment type (F), Active engagement with nature (F) Safety concerns (B), Staff attitudes and lack of staff education and awareness (B), Social prejudice and stigma (B), Limited staff and resources (B); weather (B/F); Negative self-perception and lack of confidence (B); Poor physical and visual access (B), Poor garden design (B) (i.e. benches, weather protection), Care culture NOT person-centred (B) (151); Environment type (F), Physical activity (F) (160); Transportation (F), Staff knowledge (F), Group organisation (F), Biodiversity (F) (165)
	Positive mood state	[140][143] [157][158] [167][169]	Positive	Environment type (F) (140); Physical activity (F), Environment type (F) (158); Conservation (F), Physical activity (F), Social interaction (F) (167)
	Negative affect	[141][159] [161][164]	Negative	Stressful life events (B), physical activity (F) (141); Environment type (forests>roadside> daily activities) (F), Physical activity (F) (159); Environment type (ocean>pool) (F), Biodiversity (threatening) (B) (161); Environment type (F) (164)
	Mood disturbance	[143]	Negative	Seasons – Spring (F) (143)
	Self-esteem	[139][151] [158][162] [168]	Positive	Gender – more effect for women vs men (F), Duration of intervention (F) (139); Environment type (F), Active engagement with nature (F), Safety concerns (B), Staff

**Emotional Health**				attitudes and lack of staff education and awareness (B), Social prejudice and stigma (B), Limited staff and resources (B); weather (B/F); Negative self-perception and lack of confidence (B); Poor physical and visual access (B), Poor garden design (B) (i.e. benches, weather protection), Care culture NOT person-centred (B) (151); Physical activity (F), Environment type (F) (158); Environment type (F), Individual factors (i.e. time pressures, changing identities) (B), Presence of others (B/F) (162); Access (B/F), Fear and stigma (B), Lack of resources/equipment (B) (168)
	Self-confidence	[153]	Positive	Active engagement in nature (F), Presence of others (F) (153)
	Vitality	[140][144]	Positive	Environment type (F) (140); Environment type (F), Presence of animals (F) (144)
	Agitation	[151][156]	Negative	Environment type (F), Active engagement with nature (F) (151); Garden design (F), Mobility (F), Activity itself (TH) (F) (156)
**Emotional Health**	Behavioural Problems (i.e. inattention, hyperactivity, violence)	[150][151] [157[[158]	Negative	Physical activity (F), Environment type (F) (150); Environment type (F), Active engagement with nature (F) (151); Environment type (F), Accessibility (B), Ethnicity, i.e. south Asian children (B), Deprivation (B), Quality of GS (B/F) (158); Environment type (F), Physical activity (F) (157)

	Perceived restoration	[137][138] [140][143] [160][169]	Positive	Environment type (F) < perceived sound quality (F) (137); sensory qualities (F), safety (B), topography (F) (138); Environment type (F) (140); presence of water (F) (143); Environment type (F) (160); Environment type (F) (169)
**Stress**	Perceived stress	[141][164] [165]	Negative	Stressful life events (B), physical activity (F) (141); Environment type (F) (164); Transportation (F), Staff knowledge (F), Group organisation (F), Biodiversity (F) (165)
	Psychological resistance	[132][134] [168]	Positive	Environment type (F) (132); Environment type (F) (134); Access (B/F), Fear and stigma (B), Lack of resources/equipment (B) (168)
**Stress**	Stress reduction	[132][141] [144][151] [160][159] [161][162] [167]	Positive	Environment type (F) (132); Presence of others (B), Physical activity (F) (141); Environment type (F), Presence of animals (F) (144); Environment type (F), Active engagement with nature (F) Safety concerns (B), Staff attitudes and lack of staff education and awareness (B), Social prejudice and stigma (B), Limited staff and resources (B); weather (B/F); Negative self-perception and lack of confidence (B); Poor physical and visual access (B), Poor garden design (B) (i.e. benches, weather protection), Care culture NOT person-centred (B) (151); Environment type (forests>roadside> daily activities) (F),

				Physical activity (F) (159); Environment type (F) (160); Activity itself – i.e. swimming in ocean (F) (161); Environment type (i.e. trees, plants, views, etc.) (F), Poor quality of GS/BS (B), Deprivation (B) (162); Conservation (F), Physical activity (F), Social interaction (F), Location of NBI (natural reserve>bog>field) (F) (167)
	Psychological distress	[158]	Negative	Environment type (F), Accessibility (B), Ethnicity, i.e. south Asian children (B), Deprivation (B) (158)
**Physiological Outcomes**				
Category	*Outcome*	*Reference*	*Association (with NOE)*	*Modulators (Barrier/Facilitator)*
**Cardio-vascular outcomes**	Blood pressure (systolic and diastolic)	[132][140] [143][145]	Negative/ No effects	**NEGATIVE**: Environment type (F) (132); Environment type (F), activity itself (viewing>walking) (F) (140); Environment type (urban park vs control), seasons (summer) (F/B) (143); **NO EFFECTS**: temperature (B), humidity (B), light spectrum (G/B ratio too high) (B) (145)
	Heart rate	[132][140][143] [145] [157]	Negative/ Positive	**Negative**: Environment type (F) (132); Environment type (F) – in favour of urban forests > urban parks, noise pollution (B), air pollution (B) (140) Features of the environment (trees species – where

				maple>oak>birch) (F) (145); Environment type (green>control) (F), Physical activity (F) (157)<br>**Positive**: environment type (post-viewing nature) (F) (143);
	Heart rate variability (HRV) - SDNN	[140]<br>[157][169]	Positive/<br>mixed effects	**Positive**: Environment type (F) – increased in green environment vs control (140); Environment type (F) (parks>urban) (169)<br>**No effects**: Environment type (F), Poor study design (B) (157)
**Stress**	Cortisol	[140][160]	Negative	Environment type (F) – all decrease but green (forests>park) > control (140); Environment type (F) (160)
**Physical Health Outcomes**				
Category	*Outcome*	*Reference*	*Association (with NOE or NBI)*	*Modulators (Barrier/Facilitator)*
	Physical activity (LTPA)	[133]	Positive	Safety concerns (B), presence of others (B/F), accessibility (F), natural environment (F), environmental design (B/F) (133)
	Physical activity – swimming	[134]	Positive	Fear and stigma on body type (B) (134)
	Physical activity – walking in nature	[138][154]	Positive	Proximity (F) (138); Air and heat-related pollution (B), Proximity (F), SES (B/F) (154)

**Physical activity in GS or BS**	Perceived physical health	[134][135] [146]	Positive	Physical activity in sea (F) (134); Safety (B), accessibility (B/F), personal perceptions (B/F) (135); Interpersonal processes (F), Environmental processes (B/F – based on access, perceived aesthetics, and neighbourhood attachment) (146)
	Physical activity in Urban Green Spaces (UGS)	[147][152] [160]	Positive	Changes to the built environment in parks (F), Proximity to newly built cycling/walk lanes (F) (147); Micro-features of the environment (benches) (F), Gender (F – for sedentary women), Accessibility (B) (152); Accessibility (B), Attractivity and activity in programs (B/F), Age (F) – i.e. children and young adults (160)
	Physical activity (MVPA)	[157][166]	Positive	Exposure to GS (F) (157); Duration, i.e. longer time in GS (F), Environment type (F) (166)
**Physical activity in GS or BS**	Sedentary Time	[166]	Negative	Duration, i.e. longer time in GS (F), Environment type (F) (**166**)
	Physical fitness	[168]	Positive	Environment type (F), Intervention (i.e. surfing) (F), Access (B/F), Fear and stigma (B), Lack of resources/equipment (B) (168)
**Fatigue**	Post-stroke fatigue (PSF)	[142]	Negative	Intervention (F) (142)
**Mortality**	All-cause mortality	[148] [157]	Negative	Positive affect (F), Heat reduction (F), Environment type (F) (148); Environment type (F) (157)
**General physical health**	Overall health	[150][158] [165]	Positive	Physical activity (F), Environment type (F), Accessibility (F), Quality and quantity of GS (F) (150); Physical activity (F), Environment

				type (F) (158); Transportation (F), Staff knowledge (F), Group organisation (F), Biodiversity (F) (165)
**Motor functioning**	Mobility	[152]	Positive	Micro-features of the environment, i.e. benches (F), Injury (B), Engagement in social interactions (F), Accessibility (B) (152)
	Disability	[142]	Negative	No differences between intervention vs control – both decreased (142)
**Recovery**	Recovery (from mental illnesses)	[153]	Positive	Presence of others (F), Active engagement in nature (F) (153)
**Obesity**	Obesity	[148][154] [160][165]	No effect	Quality of study design (B) (148, 154, 160)
**Sleep**	Sleep (quality and quantity)	[155]	Positive	Time of day (afternoon>morning for walking) (F), Environment type (outdoors>indoors) (F), Behavioural contexts (weekdays vs weekends preferences for different GS) (F) (155)
**Cognitive Outcomes**				
Category	*Outcome*	*Reference*	*Association (with NOE)*	*Modulators (Barrier/Facilitator)*
	Science, technology, engineering, and math (STEM)-capacity	[131]	Positive	Duration of intervention (F); stressful life events (B); environment type (F) (131)

**Cognition**	Attention Retention	[137][144] [157][160]	No effect/ Positive	**No effects** poor quality of measurements (B) (137); **Positive**: Environment type (F), Presence of animals (F) (144); Environment type (F), Physical activity (F) (157); Poor study design (B), Environment type (F) (160)
**Cognition**	Attention restoration	[132][150]	Positive	Environment type (F) (132); Physical activity (F), Proximity (F), Environment type (F), Accessibility (F), Quality and quantity of GS (F) (150)
**Symptom reduction**	ADHD symptoms	[150]	Negative	Attention restoration (F), Spatial working memory (F), environment type (F) (150)
**Memory**	Spatial working memory	[150][169]	Positive/ no effects	**Positive**: Physical activity (F), Proximity (F), Environment type (F) (150); **No effects**: (169)
	Executive functioning (I.e. memory)	[156][159]	Positive/ no effects	**Positive**: Active engagement in activity (F) (156) **No effects**: Poor study design (B), Poor measurements (B) (159)
